# Evaluation of the performance of promising host transcriptional biosignatures in predicting TB progression during a 1-year prospective cohort study in Indian household contacts

**DOI:** 10.3389/ftubr.2026.1750007

**Published:** 2026-04-17

**Authors:** Synne Jenum, Steffen Hadasch, Dhanasekaran Sivakumaran, Christina Skår Saghaug, Jonas Høgberg, Kasama Chusang Larsen, Sumithra Selvan, Helene Larsen, Christian Ritz, Harleen M. S. Grewal

**Affiliations:** 1 Department of Infectious Diseases, Oslo University Hospital, Oslo, Norway; 2 Department of Clinical Science, Faculty of Medicine, BIDS Group, University of Bergen, Bergen, Norway; 3 National Institute of Public Health, University of Southern Denmark, Copenhagen, Denmark; 4 Centre for Diagnostics, DTU Health Tech, Technical University of Denmark, Lyngby, Denmark; 5 Division of Health and Humanities, St. John's Research Institute, Bangalore, India; 6 Department of Microbiology, Haukeland University Hospital, Bergen, Norway

**Keywords:** India, INDIA11-signature, risk of progression, RISK6, Sweeney3, transcriptional biosignatures, tuberculosis

## Abstract

**Introduction:**

Achieving the goal of the End TB Strategy depends on new tools, preferably blood-based point-of-care (POC) tests, to identify individuals at risk of progression from M. tuberculosis infection to subclinical or clinical tuberculosis (TB). A decade of signature discovery and validation has resulted in several transcriptional signatures with promising capacity for predicting TB progression within the next 3–6 months, but evaluation of signature performance in Asian populations is lacking.

**Methods:**

Nested within a prospective observational cohort study of Indian household contacts (HHC study), we adapted the RISK6 and Sweeney3 along with our locally derived INDIA11 signature to the microfluidic RT-qPCR platform (Fluidigm) and evaluated their capacity to predict TB progression during a 12-month follow-up in a head-to-head comparison. As readily available in our dataset, the recently published single-gene signatures GBP2, FCGR1B, and SERPING1 were also assessed.

**Results:**

Of the 525 recruited household contacts, 12 (6 cases in children aged 5–14 years) progressed to TB (5 at 6 months, 7 at 12 months) as defined by growth of M. tuberculosis in respiratory specimens. RISK6 and Sweeney3 were successfully adapted to the Fluidigm platform. One gene in the INDIA11 assay failed, resulting in the INDIA10 signature with an overall failure rate of 5.7%. RISK6, Sweeney3, and INDIA10 demonstrated comparable but statistically non-significant predictive performance for TB progression, with AUCs: RISK6 0.61 (95%CI 0.41–0.82), Sweeney3 0.61 (95%CI 0.41–0.80), INDIA10 0.59 (95%CI 0.46–0.72). Applying a fixed value of sensitivity 75% corresponding to the WHO minimum target for TB prediction resulted in the corresponding specificities RISK6 0.49 (95%CI 0.41–0.56), Sweeney3 0.32 (95%CI 0.25–0.39), and INDIA10 0.42 (95%CI 0.35–0.50). Of single-gene signatures, the AUCs of FCGR1B and GBP2 were significant with specificities of FCGR1B 0.56 (95%CL 0.49–0.64) and GBP 0.50 (95%CL 0.43–0.58).

**Discussion:**

The expression of interferon-gamma–inducible gene signatures were able to discriminate between TB progressors and non-progressors in an hitherto underexplored Indian population, supporting the generalizability of RNA technology to this setting. Given the limited sensitivity and specificity of current biosignatures in all populations, exploration of integrated diagnostic algorithms relying on established and novel tools in a multidisciplinary approach is warranted.

## Introduction

Tuberculosis (TB), caused by the bacteria *Mycobacterium tuberculosis* (Mtb), is transmitted between people through aerosols shed by people with pulmonary TB. Worldwide, TB still causes the majority of deaths due to a single infectious agent (1). From 1994, a slow but steady decline in TB case notification rates and deaths has been achieved through extensive and systematic World Health Organization (WHO) programs. The COVID-19 pandemic has resulted in a severe set-back to the End TB Strategy wiping out ten years of achievements and estimates remains high, with of 10.7 million new cases and 1.23 million deaths in 2024 (1). As the 100-year-old BCG-vaccine confers limited and variable protection against contagious pulmonary disease, the cornerstone of TB control remains case detection, rapid treatment initiation and follow-up programs to ensure adherence throughout the treatment period of 4–15 months depending on disease severity and manifestations, and drug resistance. Identification of Mtb infected subjects at risk of TB progression for TB preventive treatment (TPT) is also a strategy endorsed by the WHO, but the coverage in people living with HIV and household contacts is well below the 90% target, with 58% and 25%, respectively (2). However, the effectiveness of the overall strategy depends on new tools, preferably blood-based point-of-care (POC) tests, to identify individuals at risk of progression (3) from Mtb infection to subclinical or clinical TB (4). Therefore, the TB scientific community has conducted extensive exploration of biomarker signatures to meet these needs for POC tests and to serve as correlates of vaccine and/or treatment efficacy to speed the pace of clinical trials (3, 5). In particular, transcriptomic (RNA) signatures are promising, and a fingerpick blood-based POC prototype has been developed (Cepheid Xpert-MTB-Host Response (HR)-Prototype cartridge system) providing a proof-of-concept that transcriptional signatures can be translated to a POC format (6–8). Several transcriptional signatures meet the target WHO accuracy benchmarks for a diagnostic triage test in symptomatic TB and for predicting TB progression within the next 3–6 months (9, 10). However, the WHO minimum target for prediction of TB progression of sensitivity and specificity ≥ 75%(11) was not met at 12 months in a recent analysis that excluded discovery datasets from the analyses (12). Notably, most signature discovery and validation for progression prediction have been mostly conducted in African populations (9, 12), therefore evaluation in other geographic and epidemiological settings is highly warranted.

Building on previous biomarker research and the availability of host RNA samples from a prospective Indian household contact cohort (HHC study), we aimed to further validate two leading transcriptional biosignatures; RISK6 (13) and Sweeney3 (14, 15) for their parsimonious application as prognostic tools along with our locally derived INDIA11-signature (16) in a head-to-head comparison on the same microfluidic RT-qPCR platform (Fluidigm). Herein we assessed the ability of the RISK6, Sweeney3 and INDIA11 signatures to predict progression to subclinical or clinical TB within one year of exposure.

## Methods

### Study design and setting

This case-control study was nested within a prospective observational cohort study of TB household contacts (HHC-study). The study was conducted in Palamaner and Kuppam Taluks, Chittoor district, Andhra Pradesh, India (3.2000°N, 78.7500°E, altitude 683 m) between September 2010 and April 2012.

### Participants

Eligible HHCs were persons living ≥75% of the time in the same household and sharing the same kitchen, out of 176 adult TB index patients diagnosed with smear positive pulmonary TB (PTB) at the microscopy centers of the Revised National Tuberculosis Control Program (RNTCP; Government of India). Contacts with previous pulmonary TB or already on TB treatment/prophylaxis were excluded. Written informed consent was given by all adults. Children aged 7–18 years gave their assents followed by parental consent whereas parental consent alone was given to children aged < 7 years.

For this nested case-control study, all concomitant TB cases, identified through the comprehensive assessments offered to all participants at baseline as described below, were excluded.

#### Baseline assessments

Assessments included a questionnaire on socioeconomic conditions, medical history, TB-symptoms and clinical examinations including weight. Mtb exposure was assessed by the Tuberculosis Contact Score (TCS)(17). The Tuberculin Skin Test (TST) and the QuantiFeron TB-Gold In-tube assay (QFT) were performed as evidence of Mtb infection. First, a blood sample was drawn for the QFT analyzed and interpreted according to the manufacturer's instructions (a positive test was defined as ≥0.35 IU/ml). Then trained staff performed a TST by injecting 2 TU Purified Protein Derivate (PPD, SPAN Diagnostics Ltd, Surat, India) intra-dermally on the volar part of the left arm. The following induration was read after 48–96 h (~80% evaluated within 72 h, the remaining within 96 h) and defined positive if ≥ 10 mm. The TST was repeated after 1–4 weeks in HHCs with an induration < 5 mm (n=54), and the baseline TST result was defined as the larger of the two tests. Two sputa (gastric aspirates for children ≤ 5 years, induced sputa for others unable to produce spontaneous sputum) were harvested on two consecutive days for smear and culture. Sputum/gastric samples were evaluated by smear microscopy for acid-fast bacilli (AFB) and cultured on both liquid (BACTEC MGIT 960^TM^ [Becton and Dickinson, USA]) and solid (Lowenstein-Jensen) media. Identification of Mtb was done using the GenoType MTBC test kit (HAIN kit). Chest X-rays were performed on all participants, anterior view for all, lateral view added on selected children < 5 years. The chest X-rays were interpreted as either “normal”, “abnormal, not TB” or “abnormal, probable TB”, first by a medical officer in the field, later by a radiologist whose interpretation was used, if evaluations were discrepant. Peripheral blood (~2.5 ml) was drawn at 0, 2, 6, and 12 months in the PAXgene Blood RNA tubes (PreAnalytiX, Hombrechtikon, Switzerland) and stored at −80°C. All HHC participants were offered HIV-testing and pre-test counseling at baseline.

#### Surveillance for TB progression

During the 1-year follow-up, HHCs were followed up with visits at 1, 2, 5, 6, 9 and 12 months, interviewed for symptoms or a suspicion or diagnosis of TB since the last visit. HHCs with ≥1 symptoms suggestive of TB were referred for diagnostic work-up including chest X-ray. Sputum (gastric aspirates for children ≤ 5 years, induced sputa for others unable to produce spontaneous sputum), one sample per participant was harvested at 6 and 12 months and examined by smear and culture as described.

#### Definition of TB progressors

Household contacts who progressed to TB were termed a TB progressor, defined by positive Mtb culture in sputum (gastric aspirates for children ≤ 5 years). Notably, this definition that does not rely on symptoms and/or radiology includes subclinical and clinical pulmonary TB cases (3). TB progressors were referred to the RNTCP center for further assessment and treatment.

#### Selection of household controls

Household contacts who remained culture-negative for Mtb throughout 12 months were classified as non-progressors. We included only contacts with paired tuberculin skin test (TST) and QuantiFERON-TB (QFT) results at baseline and 12 months to allow longitudinal stratification. Individuals with QFT reversion (from positive to negative test result) were excluded due to challenging interpretation of Mtb infection status from diverging results in low-endemic (18) and high-endemic settings (19). Three prespecified subgroups were defined: (1) **TST/QFT converters:** negative at baseline, positive at 12 months, consistent with recent infection around or after contact investigation. (2) **TST/QFT consistent positives:** positive at baseline and 12 months, consistent with infection preceding contact investigation and persistent assay positivity. (3) **TST/QFT consistent negatives:** negative at baseline and 12 months despite documented household exposure, without detectable Mtb-specific responses by these assays likely due to protective innate mechanisms.

### Transcriptional biosignatures

#### RNA isolation

RNA extraction from PAX-gene tubers was performed according to the manufacture's description (PAXgene Blood RNA kit; PreAnalytiX, Hilden, Germany). Total RNA concentration and purity were strangemeasured using a Nanodrop spectrophotometer (Thermoscientific, Wilmington, DE, USA), ranged between 0.46 −13.3 μg (average 2.8 ± 0.25μg), and had very satisfactory integrity (Agilent 2,100 Bioanalyzer).

#### Selection of transcriptional signatures and analysis platform

This study is part of our PARSISign project (*Parsimonious transcriptomic signatures for TB diagnosis and evaluation of treatment response. An evaluation in prospective Indian childhood and adult cohorts*). As the overall aim of this project was to evaluate signatures serving multiple purposes: (a) triage/diagnosis, (b) prediction of TB progression, and (c) treatment response. Two published transcriptional signatures with demonstrated potential across all these applications, and reflecting mycobacterial load or pathology were selected; **RISK6** (13) comprising the transcripts *FCGR1B, GBP2, SERPING1, SDR39U1, TRMT2A, TUBGCP6*, and **Sweeney3** (14, 15) comprising the transcripts *DUSP3, KLF2, GBP5* were chosen. In addition, we included our INDIA11 signature for subclinical and incipient TB (16), consisting of the transcripts *ABLIM2, C20orf197, CTC-543D15.3, CTD-2503016.3, HLADRB3, METRNL, RAB11B-AS1, RP4–614C10.2, RNA5SP345, RSU1P1, UACA*). We considered inclusion of our previous GJOEN7 or GJOEN10 signatures, which showed promising diagnostic potential in the Indian pediatric population (20) but these were excluded based on the poor diagnostic potential in our Indian Household contacts (21).

All gene targets belonging to the RISK6, Sweeney3 or INDIA11 signatures, along with two housekeeping genes, *B2M* and *GAPDH*, were subsequently chosen for adaptation to the Fluidigm platform (the BioMark HD Standard Biotools) and the BioMark 192.24 GE Delta Gene IFC chip). Primer and probe sequences for these 22 transcriptional targets were selected, designed using the Delta Gene^TM^ Assay design tool purchased from Standard BioTools (San Francisco, U.S); Supplementary material 1. Some of the published primer and probe sequences were modified to improve the specificity or to adapt to the selected PCR conditions. For the *HLADRB3* target (INDIA11 signature), primers were designed based on *HLADRB1* (Gene ID 3125), covering both *HLADRB3* variants. New primer and probe sequences were designed based on alignments of target gene sequences retrieved from GenBank and aligned using CLC Main Workbench (version 8.0, QIAGEN). The specificity of the oligonucleotides was tested *in silico* using nucleotide BLAST, and the melting temperatures and basic properties were approximated using OligoCalc.

#### cDNA conversion

cDNA was synthesized by performing reverse transcription of 100 ng RNA (incubation at 25°C for 5 min, 42°C for 30 min and 85°C for 5 min) applying the Reverse Transcription Master Mix (Standard BioTools, South San Francisco, CA, USA), and stored at−20°C until preamplification using a preamplification master mix consisting of Preamp Master Mix (PN 100 5581) and a Pooled Delta Gene assay mic (500 nM) and DNase-free water, all according to the manufacturer's instructions. Following assay-optimalization for the targets of interest, thermal cycling conditions were set to 95°C for 2 min followed by 14 cycles at 95°C for 15 s and 60°C for 4 min, and preamplified cDNA was diluted at a 1:10 with DNA suspension buffer (TEKnova, PN T0221).

#### High-throughput qPCR gene expression analysis on the Fluidigm platform

The expression of the selected 24 genes was measured using the BioMark 192.24 GE Delta Gene Sample and Assay Reagent Kit and IFC with ssoFast EvaGreen Supermix with Low ROX ^TM^ on the BioMark^TM^ HD system (Fluidigm, Standard BioTools) according to the manufacturer's instructions. Samples were loaded on the plates in random order with technicians blinded to the participants TB status. Each plate had two positive and one negative control. Plate to plate variation was assessed by dividing the average ct value for the positives control with each plate average, and with a correction factor ranging from 0.986 to 1.012, correction for plate-to-plate variation was not considered required.

Data was analyzed using Standard BioTools Real-Time PCR Analysis Software (version 4.8.1)

Melting-temperature (Tm) curves and cut-offs were manually inspected for all assays and samples in order to avoid nonspecific products. The assay for the CTC-543D15.3 target (FLDM-982892.1) within the INDIA11 signature failed, these data were omitted, yielding a modified INDIA10 signature. Double peaks were observed in some samples for the targets *TRMT2A* and *ABLIM2 on* the Fluidigm platform. To verify the specificity, PCR products were analyzed by agarose gel electrophoresis, followed by Sanger sequencing. The resulting sequences were confirmed using the Basic Local Alignment Search Tool (BLAST) provided by the National Center for Biotechnology Information (NCBI) (22). For TRMT2A, two distinct fragments were identified through sequencing and BLAST analysis: a specific fragment of 81-bp amplicon (intended target) and a non-specific fragment of 267-bp amplicon, the latter was excluded from analyses due to its distinct Tm and BLAST mapping to genomic DNA. For ABLIM2, a single 87-bp fragment was confirmed as the intended target through sequencing and BLAST analysis, even in samples exhibiting double peaks. These findings support the validation of the applied melting-temperature cut-offs for distinguishing specific amplicons, even in ambiguous melting profiles (Supplementary material 2).

### Statistical methods

Descriptive baseline characteristics were summarized by percentages, median with interquartile range as appropriate, and compared between TB progressors and non-progressors using Fisher's exact test (binary variables), logistic regression (multiple category variables), and Mann-Whitney test (continuous variables), as appropriate in IBM SPSS Statistics version 30.0.

The RISK6 and Sweeney3 signature scores were calculated according to previous publications. The RISK6 signature score was computed as: *score* = *geometric mean* (*GBP*2*, FCGR*1*B, SERPING*1)– *geometric mean* (*TUBGCP*6*, TRMT*2*A, SDR*39*U*1), and the Sweeney3 score as: *score* = (*GBP*5 + *DUSP*3/2) – *KLF*2, using raw CT values (9). The INDIA11 signature score was computed using previously published coefficients (16) and can be expressed as a matrix-vector product *score* = *Xb*, meaning that the score of an individual's score represents a weighted sum of the coefficients. Notably, assay failure of the CTC-543D15.3 target resulted in a modified INDIA10 signature. Furthermore, inspired by Greenan-Barret et al. (12), single-gene signatures were evaluated for some genes of the RISK6 signature, namely *GBP*2*, FCGR*1*B*, and *SERPING*1. In all score calculations, the raw CT values were used. These signatures were investigated for TB progressors and in household contacts overall as well as stratified by longitudinal TST/QFT results. Statistical analyses were performed using R (version 4.5.0)(23). The pROC package (24) was utilized to calculate area under the curve (AUC) values. To assess how well the signatures met the WHO target product profile for predicting TB progression (minimum sensitivity and specificity of 75%), sensitivity was fixed at 75% and the corresponding specificity was estimated.

## Results

### Characteristics of TB progressors and non-progressors

**Figure 1:**
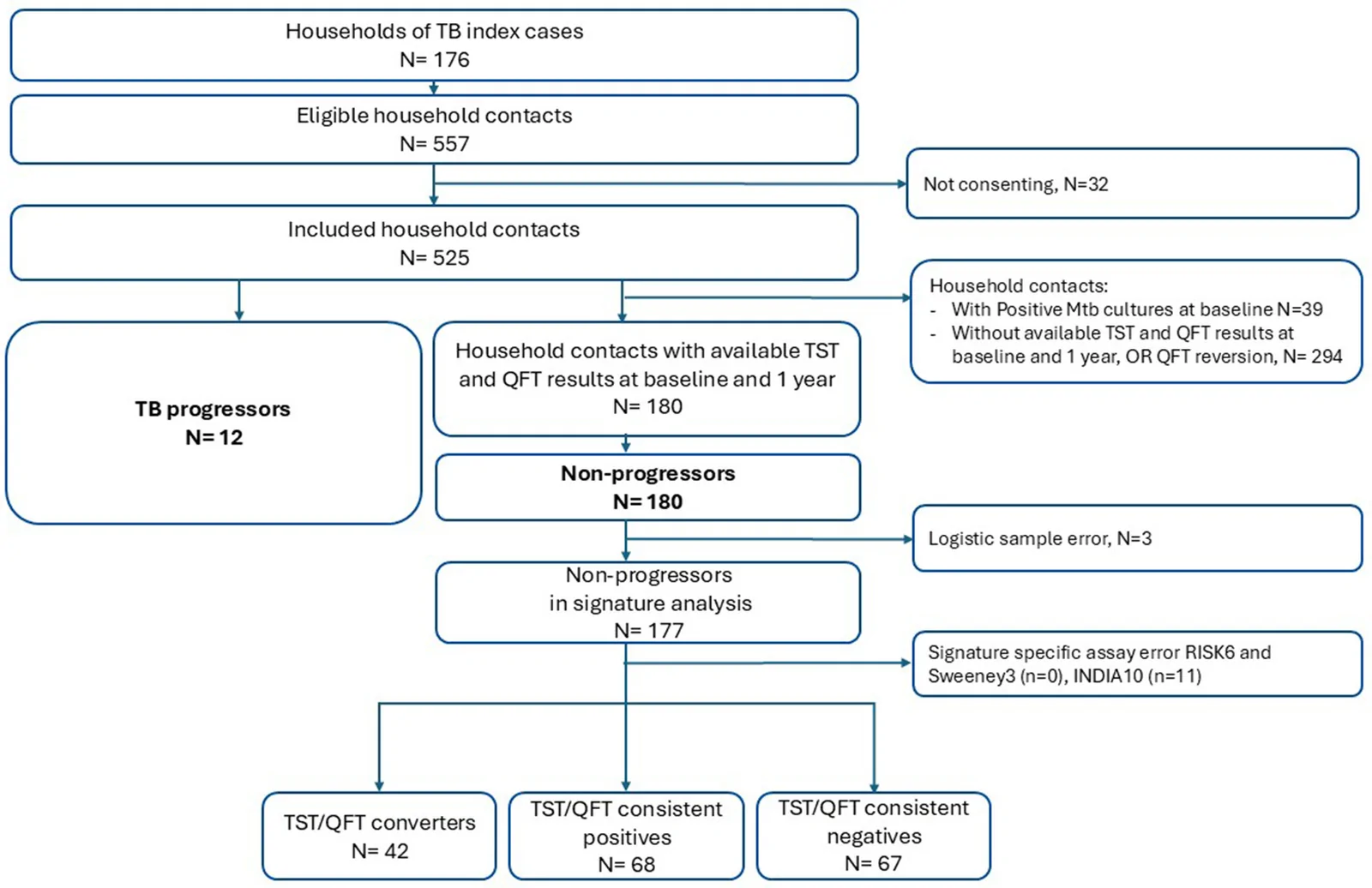
Study flow chart. Recruitment of household contacts in the HHC-study. Household contacts who progressed to TB during the 1-year follow-up were termed a TB progressors. A TB progressor was defined by positive Mtb culture in sputum (gastric aspirates for children ≤5 years). Household contacts that remained Mtb culture negative throughout the 1-year follow-up were defined as non-progressors. These were selected among household contacts with available TST and QFT results at baseline and at 1-year, enabling stratification of longitudinal TST and QFT responses: TST/QFT converters denoting household contacts testing TST and QFT negative at baseline but converting to positive tests at 1 year, representative of Mtb exposure around the timepoint of contact investigation or later and mounting Mtb-specific immune responses. TST/QFT consistent positives denoting household contacts testing TST and QFT positive at baseline and 1 year, representative of Mtb exposure at any timepoint preceding the contact investigation and resulting in long-term Mtb-specific immune responses. TST/QFT consistent negatives denoting household contacts testing TST and QFT negative at baseline and 1 year despite certain Mtb exposure in the household, that do not mount Mtb-specific immune responses likely due to protective innate mechanisms.

A total of 525 household contacts (HHCs) were recruited from 176 index cases in the study area, accounting for 94.3 % of all eligible households. Twelve TB progressors were identified during the 1-year follow-up (Figure 1 and Supplementary material 3), of whom 5 had positive Mtb cultures at 6 months and 7 had positive cultures at 12 months. Six cases occurred among children and adolescents aged 5–14 years, 5 cases occurred among adults aged 40–55 years, whereas only one young adult (aged 19 years) experienced TB progression. No TB progressors were found among children < 5 years. No significant differences in gender and age distribution were observed between TB progressors and non-progressors (Table 1). TB progressors more frequently had immunological evidence of mycobacterial exposure, as measured by the TST as measured by mm (11.0 vs. 7.0 mm, *p* = 0.019) or TST ≥10 mm (58% vs. 26%, *p* = 0.040), and a positive QFT (91% vs. 39%, *p* < 0.001). Ten of 12 TB progressors were QFT positive at baseline, and six of these also had a positive TST. QFT conversion at 2 months took place in two TB progressors; a female aged 37 years (QFT indeterminate, TST 10 mm at baseline) and a female aged 51 years (QFT negative, TST 6 mm at baseline). 6 had a positive TST. No significant differences were observed with regard to the presence of BCG scar, the Tuberculosis Contact Score (17, 25, 26), or crowding. Interestingly, TB progressors were more frequently exposed to usage of Liquefied Petroleum Gas (LPG) within the household. As expected, co-morbidity with diabetes mellitus was more frequent among TB progressors. Interestingly, two TB progressors had a chest X-ray at baseline interpreted as *Abnormal, probable TB* at baseline. Both these cases had positive Mtb culture at 6 months. One progressor, a 13-year-old boy had cough for ≥2 weeks as the only symptom at baseline and was referred to the local hospital for a diagnostic TB work-up according to the study protocol, but did not receive a diagnosis of TB, and at 5 months he was asymptomatic. The second case, a 42-year-old man had no symptoms at baseline thus not fulfilling the criteria for suspected active TB, as was therefore not referred. Two TB progressors occurred within the same family, and 14 (7.8%) of non-progressors lived in households with HIV-testing was performed in 147 (76.6%) participants of whom 129 were tested at baseline and 18 at 6 months, all except one non-progressor tested at 6 months, were HIV negative.

**Table 1:** Clinical characteristics in household contacts, both secondary TB cases and non-progressors.

Clinical characteristics	All HHCs	TB progressors	Non-progressors	
	*N* = 192	*N* = 12	*N* = 180	
	*n* (%)	*n* (%)	*n* (%)	*p*-value
Demographic and socioeconomic factors				
Gender (male)	81 (42.2)	4 (33.3)	77 (42.8)	0.57
Age (years)^a^				
<5	20 (10.5)	0	20 (11.2)	0.998
5–14	61 (31.9)	6 (50.0)	55 (30.7)	0.29
>14 (reference)	110 (57.6)	6 (50.0)	104 (58.1)	
**Mycobacterial exposure**				
BCG scar present	99 (54.1)	8 (66.7)	91 (53.2)	0.55
Secondary TB case within the family	na	na	14 (7.8)	
Tuberculosis Contact Score^b^				
Median (Interquartile range)	11.5 (3)	12.5 (4)	11.0 (3)	0.23
Tuberculin Skin Test (TST)				
mm (median, Interquartile range)	8.0 (4)	11.0 (9)	7.0 (4)	*0.02*
≥10 mm	54 (28.3)	7 (58.3)	47 (26.3)	*0.04*
Quantiferon (QFT)				
≥0.35IU/mL^c^	78 (42.4)	10 (90.9)	68 (39.3)	* <0.001*
Indeterminate	4 (2.1)	1 (8.3)	3 (1.7)	* <0.001*
Environmental risk factors				
Crowding^d^ (median, interquartile range)	3.8 (3.1)	3.5 (1.4)	4.0 (3.4)	0.74
In-door pollution^e^				
LPG	26 (14.9)	4 (40.0)	22 (13.4)	*0.04*
Kerosene	9 (5.2)	0	9 (5.5)	0.999
Wood + agric. Residue (reference)	139 (79.9)	6 (60.0)	133 (81.1)	–
Individual risk factors				
Hiv test performed, all negative	129 (67.2)	7 (58.3)	122 (67.8)^*^	0.53
Diabetes mellitus	5 (2.6)	2 (16.7)	3 (1.7)	*0.03*
Smoking (current and previous)	41 (21.4)	3 (25.0)	38 (21.1)	0.72
Alcohol >1 unit per week past 6 months	6 (3.1)	1 (8.3)	5 (2.8)	0.33
Symptoms or signs of TB				
Baseline	–	–	–	–
Symptoms^f^	5 (2.6)	1 (8.3)	4 (2.2)	0.28
X-ray, Abnormal, probable TB	2 (1.0)	2 (16.7)	0	* <0.001*
Follow-up symptoms^f^				
Any symptom from 1–12 months	7 (3.6)	2 (16.7)	5 (2.8)	0.063

^a^WHO based age categories.

^b^Tuberculosis Contact Score (11, 13, 14).

^c^Altogehter 8 missing/indeterminate results excluded.

^d^Number of household members divided on rooms in the house.

^e^By type of fuel: LPG, Kerosene, Wood + agricultural residue.

^f^One or more of the following defined TB symptoms: Cough > 2weeks, fever, chest pain, haemoptysis or weight loss.

^*^One non-progressor tested HIV positive at 6 months. *P*-values demarking significant differences between groups are marked in italic.

Only five non-progressors, three children (3, 7 and 10 years), and two adults (19 and 25 years) had ≥1 TB symptom from the 1 month throughout the 12 month follow-up. All were referred to diagnostic work-up where additional chest X-ray was normal, and fulfilled the follow-up without further reporting of symptoms, diagnosis or treatment of TB.

### Gene signature performance on the Fluidigm platform

Twenty one out of 22 gene targets performed well on the Fluidigm platform. The assay for the CTC-543D15.3 target (FLDM-982892.1) within the INDIA11 signature failed; these data were omitted, resulting in a modified INDIA10 signature. Its omission here constitutes a technical platform limitation rather than biological non-reproducibility. Calculation of the INDIA10 score required results for all 10 targets, which led to exclusion of 11 subjects and a signature failure rate of 5.7%.

Quality control of the Fluidigm gene-expression dataset revealed two duplicate participant IDs with discordant results. The cause could not be determined, and these subjects were excluded from further analyses.

### Prognostic performance of transcriptional signatures for TB progression in household contacts

**Figure 2:**
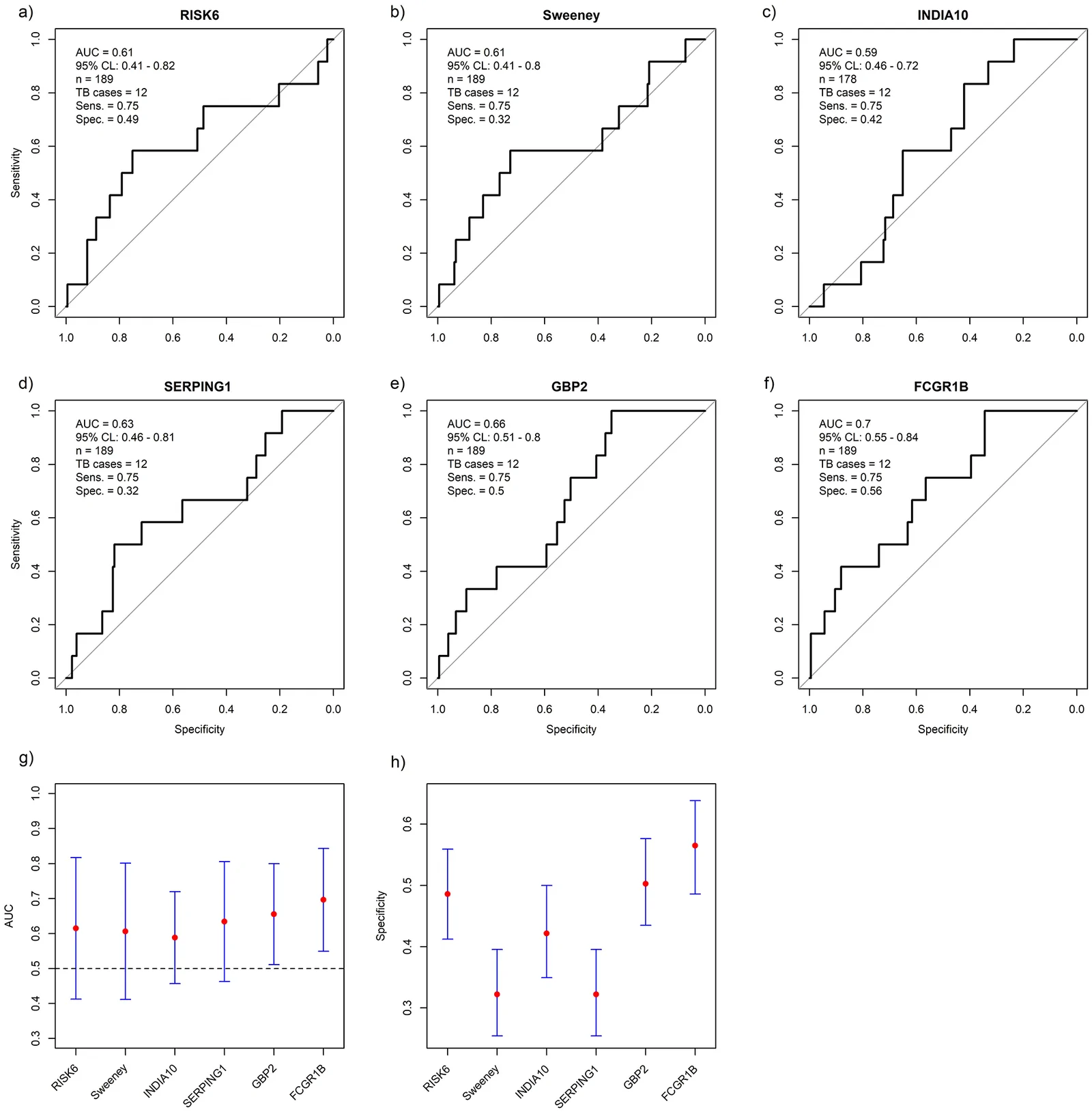
Depicted receiver operating characteristic (ROC) curves for the multiple-gene signatures RISK6 **(a)**, Sweeney3 **(b)**, INDIA10 **(c)**, and the single-gene signatures SERPING1 **(d)**, GB2 **(e)** and FCGR1B **(f)** in identifying TB progressors versus non-progressor household contacts. The total number analyzed, the number of TB progressors, and the 95%CIs are given in the figures. **(g)** Head-to-head AUC estimates with 95%CI intervals for all biosignatures. **(h)** Specificity and its 95%CIs for sensitivity=0.75.

First, in whole blood samples harvested at the timepoint of contact investigation (baseline), we investigated the capacity of the three selected transcriptional biosignatures RISK6 (13), Sweeney3 (14, 15) and the INDIA10 signature (16), to predict TB progression in household contacts with significant TB exposure, within 1 year from the contact investigation. All three showed poor prognostic performance, i.e., limited ability to distinguish contacts who later developed TB progression from non-progressors: AUCs were ~0.60 with lower 95% CI bounds < 0.50; specifically, RISK6 AUC of 0.61 (95%CI 0.41–0.82), Sweeney3 AUC of 0.61 (95%CI 0.41–0.80) and INDIA10 AUC of 0.59 (95%CI 0.46–0.72); Figures 2a–c. For the single-gene signatures, the AUC values were slightly higher; SERPING with AUC of 0.63 (95%CL 0.46–0.81), GBP with AUC of 0.66 (95%CL 0.51–0.80), and FCGR1B with AUC of 0.7 (95%CL 0.55–0.84); Figures 2d–f.

At the predefined fixed sensitivity of 75%, Sweeney and SERPING1 showed the lowest specificity, both with 0.32 (95%CL 0.25–0.39). The highest values were found for RISK6, GBP2, and FCGR1B with 0.49 (95%CL 0.41–0.56), 0.50 (95%CL 0.43–0.58), and 0.56 (95%CL 0.49−0.64), respectively (Figures 2g–h).

### Prognostic performance of transcriptional signatures for TB progression in household contacts stratified by longitudinal TST/QFT results

**Figure 3:**
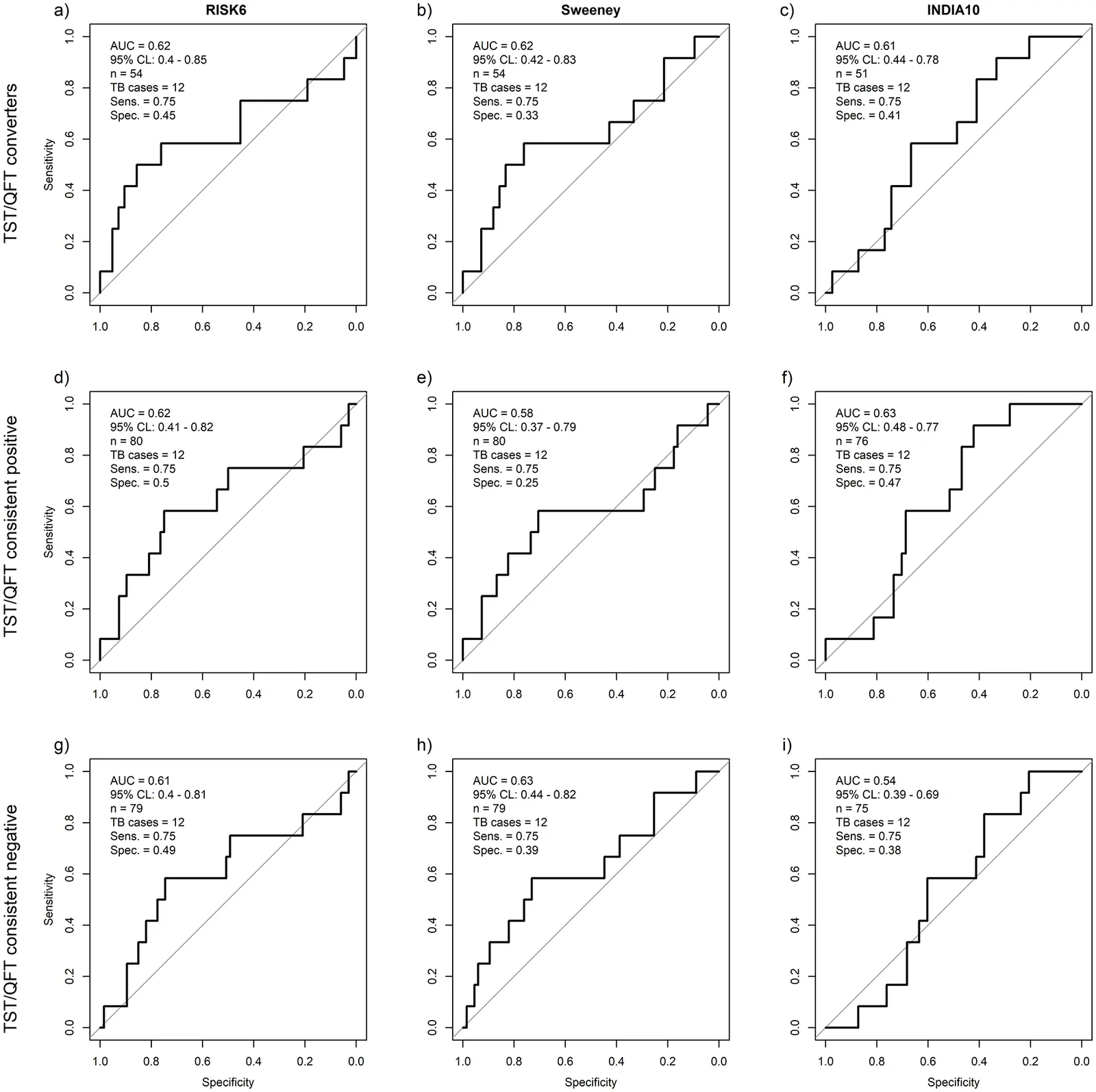
Depicted receiver operating characteristic (ROC) curves for RISK6 **(a, d, g)**, Sweeney3 **(b, e, h)**, INDIA10 **(c, f, i)** in identifying TB progressors versus non-progressor household contacts stratified for longitudinal TST and QFT results: TST/QFT converters (TST and QFT negative at baseline and positive at 1 year), TST/QFTconverters (TST and QFT negative at baseline and positive at year), **(d–f)** TST/QFT consistent positives (TST and QFT positive at baseline and at year), and **(g–i)** TST/QFT consistent negatives (TST and QFT negative at baseline and at year). Specificity estimates and confidence limits given a sensitivity of 0.75 for the different signatures.

**Figure 4:**
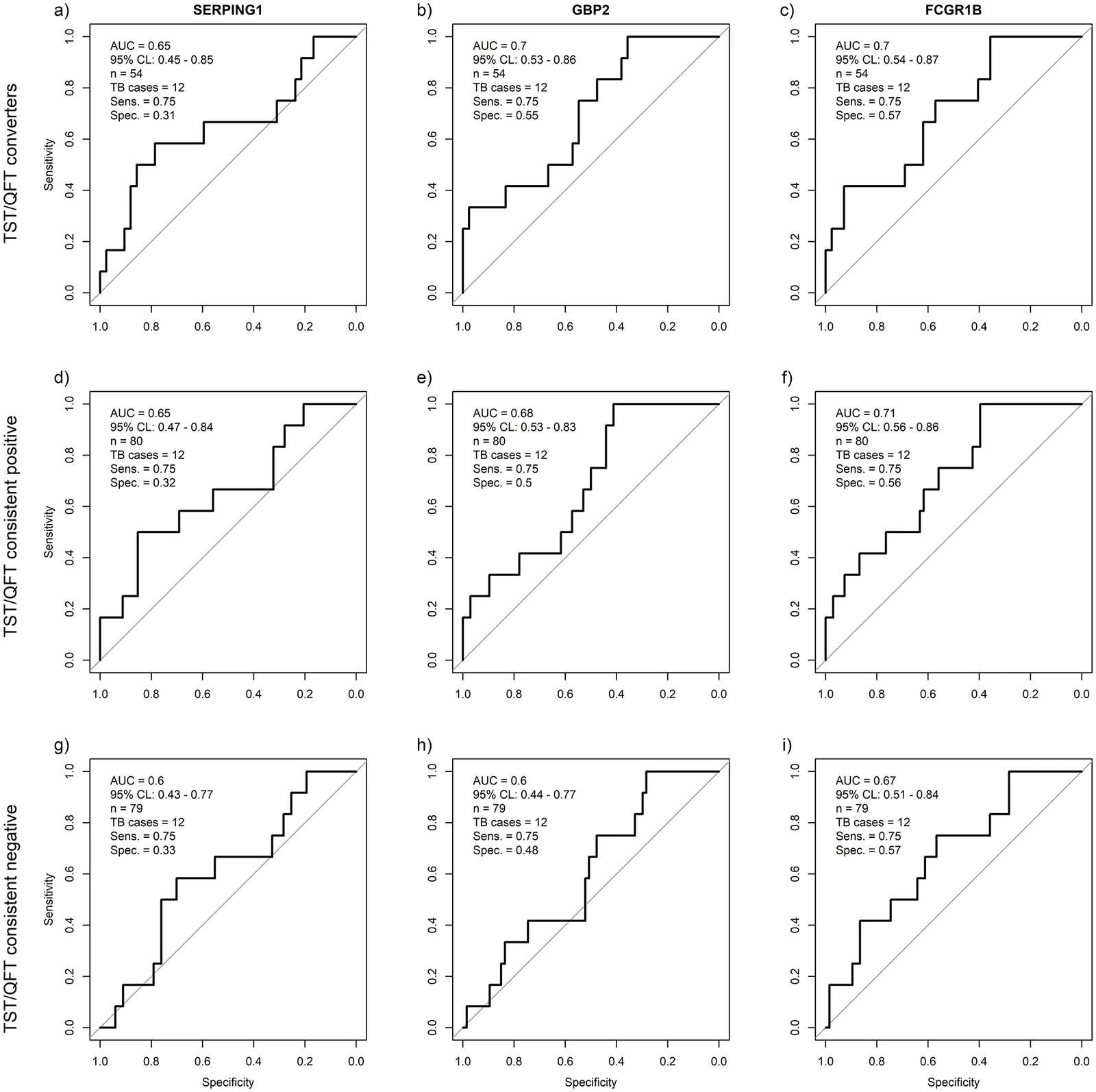
Depicted receiver operating characteristic (ROC) curves for single gene signatures SERPING1 **(a, d, g)**, GBP2 **(b, e, h)**, FCGR1B **(c, f, i)** in identifying TB progressors versus non-progressor household contacts stratified for longitudinal TST and QFT results: TST/QFTconverters (TST and QFT negative at baseline and positive at year), **(d–f)** TST/QFT consistent positives (TST and QFT positive at baseline and at year), and **(g–i)** TST/QFT consistent negatives (TST and QFT negative at baseline and at year). Specificity estimates and confidence limits given a sensitivity of 0.75 for the different signatures.

**Figure 5:**
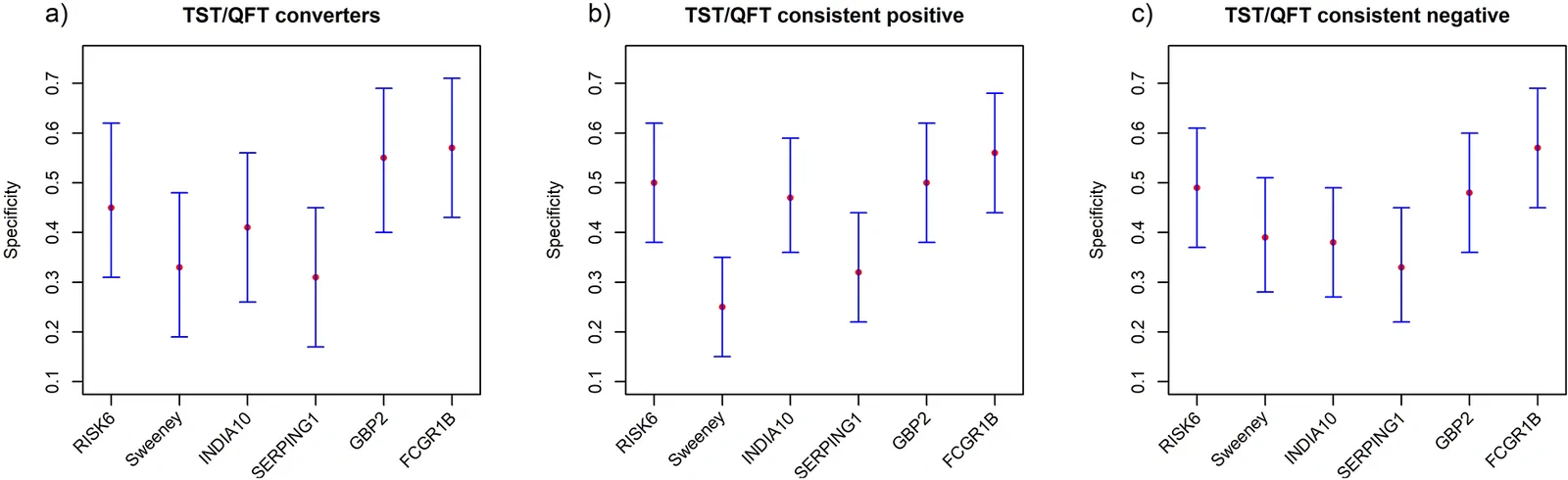
Head-to-head estimates of specificity and its 95% CIs for sensitivities fixed at 0.75 for all biosignatures in TB progressors versus TST/QFT converters **(a)**, TST/QFT consistent positives **(b)** and TST/QFT consistent negatives **(c)**.

Next, we explored the impact of mycobacterial exposure and immunity on the prognostic performance of the RISK6, Sweeney3 and INDIA10 signatures in sub analyses. Household contacts were stratified by longitudinal TST and QFT responses as described above. We again assessed each biosignature's ability at baseline to distinguish contacts who later progressed to TB compared to the subgroups TST/QFT converters, TST/QFT consistent positives and TST/QFT consistent negatives. The performance of all multi-gene signatures was equally poor across all subgroups with AUCs ranging from 0.54 to 0.63 (Figure 3). For the single-gene signatures, the AUC values with the subgroups ranged from 0.6 to 0.71, whereas the confidence limits indicated high uncertainty with lower limits between 0.43 and 0.56 (Figure 4). With the predefined fixed sensitivity of 75%, and similar to the analyses across all subgroups, RISK6, GBP2, and FCGR1B showed slightly higher specificities than the other signatures, whereas the confidence intervals overlapped in most cases (Figure 5).

Furthermore, whereas specificities for single-gene signatures were consistent across all TST/QFT subgroups, specificities for multi-gene signatures seemed to have some variation across subgroups: Sweeney3 showed poorer specificity in TST/QFT consistent positives (0.25) compared to TST/QFT consistent negatives (0.39), whereas the opposite was the case for INDIA10 performing better in TST/QFT consistent positives (0.47) compared to TST/QFT negatives (0.38), but also here, confidence intervals are overlapping.

## Discussion

This study is the first to assess the predictive capacity of the RISK6 and Sweeney3 transcriptional signatures for TB progression in an Indian population. In the prospective CORTIS-01 community cohorts of adults in South Africa, these signatures, implemented on the microfluidic real-time quantitative PCR (RT-qPCR) Fluidigm platform showed strong predictive performance within 6 months. However, further validation in diverse geographic and epidemiological settings is required to establish their global applicability for identifying high-risk individuals for targeted preventive or curative TB treatment. This gap motivated us to adapt these signatures, along with our INDIA11 signature, to the Fluidigm platform and evaluate their prognostic performance in a prospective Indian household-contact cohort. Both RISK6 and Sweeney3 were successfully adapted without assay failure. One INDIA11 gene assay failed, resulting in the INDIA10 signature with a 5.7% overall failure rate. Inspired by the promising single-gene signatures Greenan-Barret et al. (12) published after this study was planned, we also took the opportunity to evaluate *GBP*2*, FCGR*1*B*, and *SERPING1* for which data were available (three of six genes within the RISK6 signature) for the first time in an Indian population.

All three signatures: RISK6, Sweeney3, and INDIA10, demonstrated comparable but statistically non-significant predictive performance for TB progression within 12 months, with AUCs ranging from 0.59 to 0.61. In order to facilitate comparison of performance between signatures, we chose a fixed value of sensitivity 75% corresponding to the WHO minimum target for prediction of TB progression (11), and calculated the corresponding specificity; RISK6 0.49 (95%CL 0.41–0.56), Sweeney3 0.32 (95%CL 0.25–0.39), and INDIA10 0.42 (95%CL 0.35–0.50). The assay failure rate on the Fluidigm platform was low, consistent with previously reported failure rates of 1.8% for RISK6 and 5.3% for Sweeney3 in HIV-negative adults using similar RT-qPCR assays (8). The slightly higher failure rate for INDIA10 likely reflects increased complexity with a larger number of gene targets.

Whereas RISK6 and Sweeney3 have previously proved a capacity to predict progression to TB within 6 months (AUC_RISK6_ 0.91, 95%CI 0.86–0.97, AUC_Sweeney3_ 0.80, 95%CI 0.42–0.85) in community recruited adults in South-Africa, the predictive capacity in this study appeared to weaken with time and was no longer within the WHO minimum target product profile (TTP) of ≥75 sensitivity and specificity (10) at 12 months for RISK6 (AUC_RISK6_ 0.80, 95%CI 0.65–0.95, sensitivity 0.66, specificity 0.85) or Sweeney3 (AUC_Sweeney3_ 0.63 95%CI 0.42–0.85, sensitivity 0.45, specificity 0.79) (8). Whereas RISK6 performed poorer in our Indian cohort, Sweeney3 had comparable estimated performance, but the uncertainty in our study was larger due to smaller sample size (total number of participants of 192 compared to approx. 2,500) as reflected by large CIs in our study. Children ≤ 14 years constituting 32% in our cohort compared to all adults is a likely explanation in addition to geographical differences, epidemiological context of TB, co-infections and malnutrition.

Interestingly, in a recent publication by Greenan-Barrett et al. (12), the capacity of RISK6 and Sweeney3 along with other transcriptional signatures and single-gene transcripts, were evaluated again in pooled data of RNA sequencing and quantitative PCR data from approximately 5,000 participants from South- Africa, Gambia, Ethiopia, Brazil and UK, applying more stringent data analysis including leaving out the discovery dataset in the evaluation of each signature. This resulted in following performances for predicting TB disease within 0–12 months: RISK6 with AUC 0.74 (95%CI 0.69–0.77), sensitivity of 0.62 and specificity of 0.76, Sweeney3 with AUC of 0.72 (95%CI 0.68–0–77), sensitivity of 0.49 and specificity of 0.88 (12), thus neither meeting the WHO minimum TTP for TB prediction. We report a similar pattern of signature performance, with better performance for single-gene than multi-gene signatures, but AUC values were 0.1 lower in our study. Of single-gene signatures in our study, FCGR1B and GBP2 had the highest AUC values (AUC_FCGR1B_ 0.7, AUC_GBP2_ 0.66) with significant discriminatory capacity, that at a fixed sensitivity of 75%, had a specificity estimate of 0.56, with uncertainty (95%CL 0.49–0.64). The lower discriminatory performance observed in our cohort probably relates to important differences in case composition and study design compared with the meta-analysis. In the primary analysis of the meta-analysis, approximately 60% of cases represented either prevalent TB (including both symptomatic and asymptomatic disease) or TB progressors within the first 0–6 months after sampling, whereas in our cohort, prevalent cases more likely to have more advanced disease and probably more prominent interferon-gamma responses, were excluded. Only 42% developed TB within six months, and four of the six early progressors were aged ≤ 13 years. Children ≤ 14 years constituted 32% of our study population, compared with < 25% of participants below 21 years in the meta-analysis dataset, which may be relevant given known age-related differences in host immune responses and transcriptomic profiles. In addition, the meta-analysis included large nested case–control cohorts (ACS and GC6–74), in which TB progressors represented 18.7% and 23.7% of sampled participants, respectively, and together accounted for 59% of all subclinical cases included in the pooled analysis. Overestimation of performance is a well-known risk of case-control design due to disease spectrum bias. In contrast, TB progressors represented only 6.25% (12/192) of participants in our cohort. Across the full meta-analysis, subclinical cases represented 4.3% (283/6544) of participants, with a very small proportion of symptomatic prevalent disease. These differences in age distribution, timing of progression, and sampling structure are likely to contribute to divergent performance estimates and highlight the importance of validating transcriptional signatures in truly prospective, population-based cohorts.

Ideally, we should have performed stratified analyses for the signature's prognostic potential at 6 and 12 months, as the prognostic capacity at 6 months is strong for many transcriptional signatures (9). Of 12 secondary TB cases in our study, 6 were diagnosed at 6 months and 6 at 12 months, and these numbers are too small for meaningful secondary analysis, thus we might not been able to discover a capacity by all signatures to predict TB progression within 6 months.

We also conducted analyses where non-progressor household contacts were stratified by TST/QFT results, and similar AUC values were reported across TST/QFT subgroups indicating signature performance independent of Mtb infection status in non-progressor household contacts. TST and QFT tests are well-established surrogates for Mtb infection and reflect host-specific Mtb (QFT) and mycobacterial (TST) immunity following stimulation *in vitro* (QFT) or *in vivo* (TST). It is likely that presence of Mtb infection also impacts on host transcriptional biosignatures that are examined without added stimulation, thus reflect activation of innate and adaptive immune responses in the living subject. A priori, it is reasonable to assume that the discriminatory power of host transcriptional signatures would be highest between the most heterogeneous populations, notably between TB progressors and Mtb uninfected household contacts. From our results this does not seem to be the case as the pattern for both multi-gene or single-gene were very similar across TST/QFT groups, maybe with the exception of INDIA10 with better estimates for TST/QFT consistent positives compared to TST/QFT converters and TST/QFT consistent negatives, but overlapping confidence intervals limits interpretation.

Among the tested signatures, the risk of overestimation is higher for INDIA10 due to inclusion of participants from the INDIA11 discovery cohort. Notably, the INDIA10 signature performed much poorer in this study than the original INDIA11 signature (16) is likely multifactorial. In the original study, the INDIA11 signature was derived from RNA-Seq data by comparing subclinical TB and incipient TB cases within the same Indian household-contact cohort. In that study, INDIA11 demonstrated excellent ability to discriminate subclinical TB from household controls, with an AUC of 0.99 (95 % CI 0.96–1.00), sensitivity of 1.0 (95 % CI 0.74–1.0) and specificity of 0.85 (95 % CI 0.70–0.91). The *CTC-543D15.3* transcript, a low-abundance long intergenic non-coding RNA carrying a strong positive coefficient (+1.78) in the original LASSO model fit, failed on the Fluidigm platform and was therefore excluded. Its omission likely weakened the composite signal of the signature, and adaptation to the RT-qPCR system without retraining the model may have introduced scaling bias. Finally, the original case–control discovery design likely overestimated performance, whereas prospective testing probably revealed the true, more modest predictive capacity of the remaining ten-gene panel.

Common to all studies, the TB case definitions used are crucial for real-life sensitivity and specificity. Although, there is growing consensus that the identification of subclinical TB and clinical TB is equally important (4) TB case-definitions still vary across studies and are carried into meta-analyses (12). Our definition of microbiologically culture confirmed TB diagnosis in respiratory specimens regardless of the presence of signs and/or symptoms, is in line with the case definition in the CORTIS-01 cohort (9). However, we acknowledge that this approach likely misses non-infectious subclinical pulmonary cases, as well as extrapulmonary and pediatric cases where the TB diagnosis mostly relies on symptoms and signs. As pointed out earlier, the proportion of children in our study was considerably higher than previous studies. In order to minimize the misclassification of pediatric probable TB cases potentially leading to reduced specificity for the investigated biosignatures, our HHC included a thorough symptom-focused follow-up, with subsequent referral for additional chest X-ray. This limits the likelihood of un-diagnosed/wrongly classified TB progressors among HHCs included in this study. Furthermore, the low HIV prevalence in our setting is likely to reduce the burden of paucibacillary TB compared with South Africa. Such misclassifications of TB cases could have led to an underestimation of specificity.

Further limitations were the restricted selection of household contacts. Ideally, all non-progressors would have been included irrespective of TST/QFT results. This constraint arose because the PARSISign project relied on preselected samples from prior biomarker studies in the same Indian cohort (16, 21). Applying a broader borderline zone for QFT positivity (e.g. 0.20–0.70 IU/ml) to define more stringent conversion/reversion criteria was not undertaken to simplify generalization to clinical settings with standard reporting of results. We acknowledge though, that conversion/reversion close to the cut-off could in part represent assay variability.

## Conclusions and future perspectives

Early case identification remains essential to reduce TB transmission and morbidity (3). Nearly half of all TB cases are asymptomatic (24), underscoring the need for systematic surveillance of individuals recently exposed to *M. tuberculosis*. Household contact investigation following the detection of contagious index cases offers a practical and effective framework for early case finding (25) and initiation of TB preventive treatment to household contacts at risk of TB progression (2). Extensive international efforts have sought to identify biomarkers and biosignatures that predict progression from infection to active disease (3, 27), and biosignature-guided TB preventive treatment has been piloted (28), but optimal treatment strategies across the TB disease spectrum remains elusive (4). Multiple transcriptional signatures predictive of TB progression have now been identified and validated across large cohorts in Africa, Brazil, and the United Kingdom (12). Our findings, generated in an underexplored Indian population of adults and children, support the generalizability of interferon-gamma–inducible gene signatures (10) across diverse settings (9, 12) and demonstrate the feasibility of translating these signatures into point-of-care formats (6, 7). However, there is increasing recognition that their prognostic accuracy is time-limited, typically strongest within six months of sampling (9, 12, 29). Sustained progress in early case identification will therefore require structured longitudinal follow-up of high-risk individuals, particularly in low- to moderate-incidence regions where contact investigation can be systematically implemented.

Given the limited sensitivity and specificity of current biosignatures, exploration of integrated diagnostic algorithms relying on established and novel tools in a multidisciplinary approach is warranted. Early identification of contagious cases is essential to reduce transmission and should be first priority with digital chest radiography (30) and non-sputum microbiological methods, such as tongue swabs for Mtb detection using the Xpert MTB/RIF Ultra assay (31, 32), as easily deployable screening tools. Second, combining immunological assays such as TB-IGRA (12) and transcriptional single or multi-gene biosignatures (9, 12) with high sensitivity as “rule-in” tools, with biosignatures with high specificity, could improve targeted treatment of early TB disease stages. While further validation of host-response biomarkers in Asian populations is needed, future research should prioritize development of evidence-based diagnostic algorithms for early TB case detection and individualized treatment strategies spanning the subclinical-to-clinical disease spectrum.

## Data Availability

TB progressors and non-progressors are available at https://doi.org/10.5281/zenodo.19232452. The databases files from the Indian Household Contact Cohort (HCC) contains detailed data on socioeconomic status, living conditions, familie members with gender and age as well as comorbidites and HIV-status, and cannot be made publicly available for due to GDPR compliance issues.
