# Supplementary material for: Evaluation of the performance of promising host transcriptional biosignatures in predicting TB progression during a 1-year prospective cohort study in Indian household contacts
Source: Front Tuberc. 2026 Apr 17;4:1750007. doi: 10.3389/ftubr.2026.1750007 (PMC13494970; doi:10.3389/ftubr.2026.1750007)
Supplement: Supplementary file 1 [file Data_Sheet_1.pdf]

Supplementary material 1 Overview of 22 transcriptional targets covering 3 transcriptional signatures, followed by detailed information for each target including full name, function, isoforms, amplicon length and Blast hits.

Signature	Gene Target	Assay ID	Assay Name	FP	RP
INDIA11	ABLIM2	GEP00124241	ABLIM2_124241_i7	AGCAGCCAGAACTGAAGACA	GACCCTGAGGTGCTGGAA
HKG	B2M	GEP00055772	B2M_55772_i0	TTAGCTGTGCTCGCGCTAC	CTCTGCTGGATGACGTGAGTAA
INDIA11	C20ORF197	GEP00124242	C20ORF197_124242_e0	GGTGGAGGTGTCCTGCAT	GCGCTGCTGGGTGTTG
Sweeney3	DUSP3	GEA00017164	DUSP3_17164_i0	GTCACCCCGCGGATCTAC	CAGCACATGGGTGATGCCTA
RISK6	FCGR1B	GEA00016283	FCGR1B_16283_e0	CAGATTTCACTACTCCCACCAG	CCAAAGGAGCAGAGTTGTCA
INDIA11	RNA5SP345	GEP00124252	FLDM-278021_124252_e0	CCCTGAATGCACCCATCTCA	TCTTCTCCCATCCAAGTGCTAAC
INDIA11	RSU1P1	GEP00124255	FLDM-838169_124255_e0	CTGCCAACACAGATCAGCAGGCTTCAA	AGCTGGCAGGGAGCCAAAGCC
INDIA11	RP4-614C10.2	GEP00124253	FLDM-957085_124253_t-1	CCACTCCCCAGCTGTGAAA	CCATGCTGGGCAGCTCTA
INDIA11	CTD-2503O16.3	GEP00124250	FLDM-968540_124250_e0	TGGCTCTTCCTCAGGGTCTA	GTTCAGGCATTTCCACTTCTCC
INDIA11	CTC-543D15.3	GEP00124249	FLDM-982892_124249_t-1	GTGGGTGCGGAGGTATCAA	GCATGGAACAGGCTTTATCCC
HKG	GAPDH	GEP00093699	GAPDH_93699_i3	GAGAACGGGAAGCTTGTCATCA	TGGACTCCACGACGTACTCA
RISK6	GBP2	GEP00061748	GBP2_61748_i9	CCCAGTTAATGGCAGAGCAA	TCTCGAATCCCTCCTTGAGAA
Sweeney3	GBP5	GEP00058588	GBP5_58588_i2	ATGAACAAGCTGGCTGGGAA	CCAAATTCCCTTGGTGTGAGAC
INDIA11	HLA-DPB1	GEP00058599	HLA-DPB1_58599_i0	TGGCTCTGACGGCGTTAC	GTCCCATTAAACGCGTAGCA
Sweeney3	KLF2	GEP00070436	KLF2_70436_i1	GGCTGCGGCAAGACCTA	CGTCCCAGTTGCAGTGGTA
INDIA11	METRNL	GEP00124238	METRNL_124238_i2	GCTTCCAGTACGAGCTGGTTA	GAAGTCGCTGGTGCAGAC
INDIA11	RAB11B-AS1	GEP00124239	RAB11B-AS1_124239_e0	CGGCGGTTGAATGGCCTA	ACCCTCAGAGCACTACTTCCA
RISK6	SDR39U1	GEP00123270	SDR39U1_123270_i4	CCCAGGAGGGGACTTTGAC	CCACGGCCCAGCACAA
RISK6	SERPING1	GEP00055703	SERPING1_55703_i6	CCAGCCAGGATATGCTCTCAA	TGTCAGCCCACACAGGTTAA
RISK6	TRMT2A	GEP00123268	TRMT2A_123268_i3	CATCCCCGAAGCCACCAA	TCTGGGTCGTATGCCGAGTA
RISK6	TUBGCP6	GEP00124243	TUBGCP6_124243_i3	CCGTCCTGGACTCTTTGTAC	ATACTGCAGGTACCTCCTCA
INDIA11	UACA	GEP00124240	UACA_124240_i3	GGACATGCATTGTGCCTACA	ATCGTGAAGTGCAGTTCTTCC

Delta Gene Assay Report

Panel Name	22433_FDGP_23.O2
Plate ID	22433FDGP23O2_P01

A1

**Assay Name**	ABLIM2_124241_i7		**Assay ID**	GEP00124241	
**Gene Symbol**	ABLIM2	**Entrez Gene ID**	84448	**Organism**	Homo sapiens
**Official Full Name**	actin binding LIM protein family member 2				
**Related IDs**	HGNC:HGNC:19195; Ensembl:ENSG00000163995; MIM:Objectid_id; AllianceGenome:HGNC:19195				
**Also known as**					
**GO Function**	actin filament binding; metal ion binding				
**Go Process**	cytoskeleton organization; lamellipodium assembly				
**Go Component**	actin cytoskeleton; actin cytoskeleton; cytoplasm				
**Gene Isoforms**	NM_001130083.N; NM_001130085.N; NM_001130088.N; NM_001130087.N; NM_032432.N; NM_001130086.N; NM_001286688.N; NM_001130084.N				
**Blast Isoform Hits**	NM_001130086; NM_032432; NM_001130083; NM_001130085; NM_001130084; NM_001286688; NM_001130087; NM_001130088				
**Amplicon Length**	87	**Exon**	8-9	**CDS**	Full

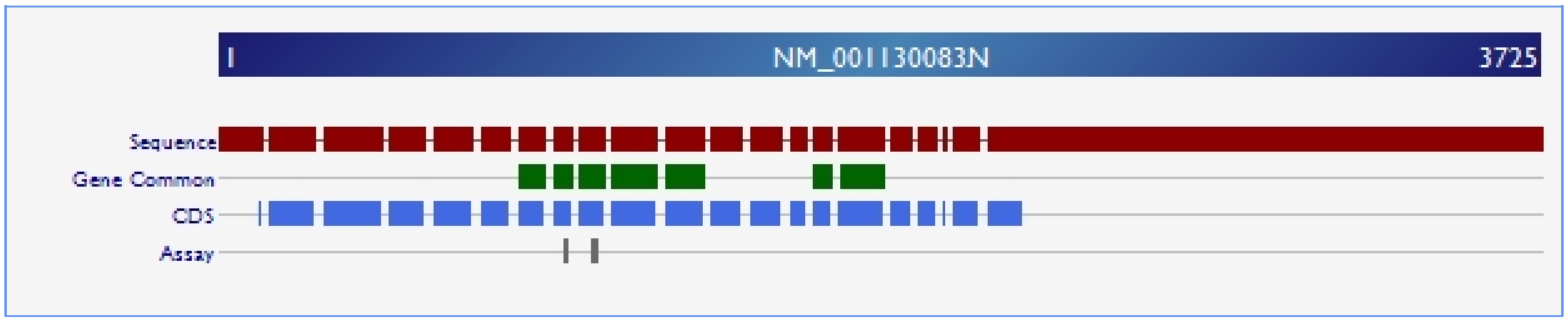

B1

**Assay Name**	B2M_55772_i0			**Assay ID**	GEP00055772
**Gene Symbol**	B2M	**Entrez Gene ID**	567	**Organism**	Homo sapiens

Official Full Name beta-2-microglobulin

Related IDs HGNC:HGNC:914; Ensembl:ENSG00000166710; MIM:Objectid_id; AllianceGenome:HGNC:914

Also known as IMD43

GO Function MHC class II protein complex binding; identical protein binding; peptide antigen binding; protein binding; protein homodimerization activity

Go Process T cell differentiation in thymus; T cell mediated cytotoxicity; amyloid fibril formation; antigen processing and presentation of endogenous peptide antigen via MHC class I; antigen processing and presentation of exogenous peptide antigen via MHC class II; antigen processing and presentation of exogenous protein antigen via MHC class Ib, TAP-dependent; cellular response to iron ion; cellular response to iron(III) ion; cellular response to nicotine; intracellular iron ion homeostasis; iron ion transport; learning or memory; learning or memory; multicellular organismal-level iron ion homeostasis; negative regulation of epithelial cell proliferation; negative regulation of forebrain neuron differentiation; negative regulation of neurogenesis; negative regulation of neuron projection development; negative regulation of receptor binding; peptide antigen assembly with MHC class II protein complex; positive regulation of T cell activation; positive regulation of T cell cytokine production; positive regulation of T cell mediated cytotoxicity; positive regulation of cellular senescence; positive regulation of ferrous iron binding; positive regulation of immune response; positive regulation of protein binding; positive regulation of receptor binding; positive regulation of receptor-mediated endocytosis; positive regulation of transferrin receptor binding; protein homotetramerization; protein refolding; regulation of erythrocyte differentiation; regulation of iron ion transport; response to molecule of bacterial origin; retina homeostasis; sensory perception of smell

Go Component ER to Golgi transport vesicle membrane; Golgi apparatus; Golgi membrane; HFE-transferrin receptor complex; MHC class I peptide loading complex; MHC class I protein complex; MHC class I protein complex; MHC class II protein complex; cytosol; early endosome lumen; early endosome membrane; endoplasmic reticulum lumen; external side of plasma membrane; extracellular exosome; extracellular region; extracellular space; focal adhesion; late endosome membrane; lysosomal membrane; membrane; phagocytic vesicle membrane; plasma membrane; plasma membrane; recycling endosome membrane; specific granule lumen; tertiary granule lumen

Gene Isoforms NM_004048.

Blast Isoform Hits NM_004048

**Amplicon Length**	90	**Exon**	1-2	**CDS**	Full

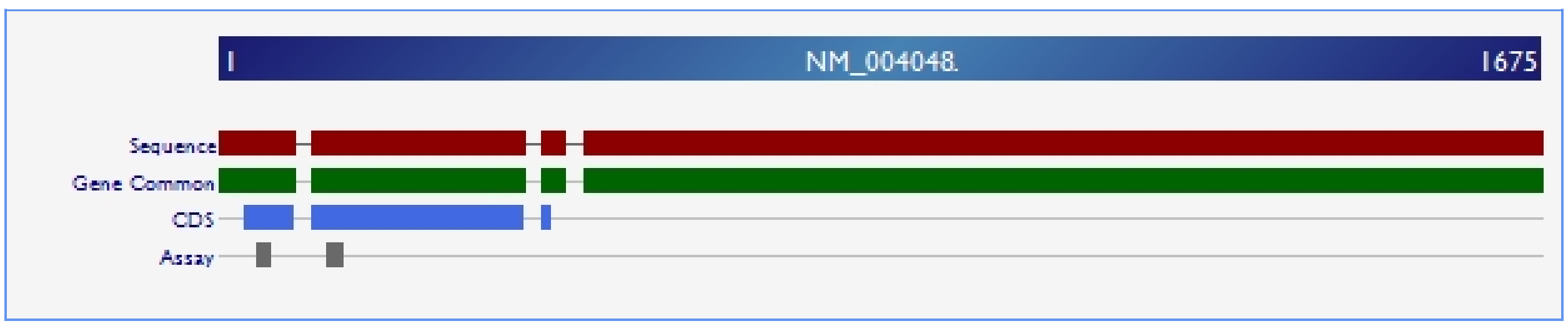

C1

**Assay Name**	C20ORF197_124242_e0			**Assay ID**	GEP00124242
**Gene Symbol**	C20ORF197	**Entrez Gene ID**	284756	**Organism**	Homo sapiens

Official Full Name long intergenic non-protein coding RNA 2910

Related IDs HGNC:HGNC:26601; Ensembl:ENSG00000176659; AllianceGenome:HGNC:26601

Also known as C20orf197

GO Function

Go Process

Go Component

Gene Isoforms NR_126504.N; NR_161291.N; NR_161292.N; NR_161293.N

Blast Isoform Hits NR_161293; NR_161291; NR_126504; NR_161292

**Amplicon Length**	60	**Exon**	1	**CDS**	Full

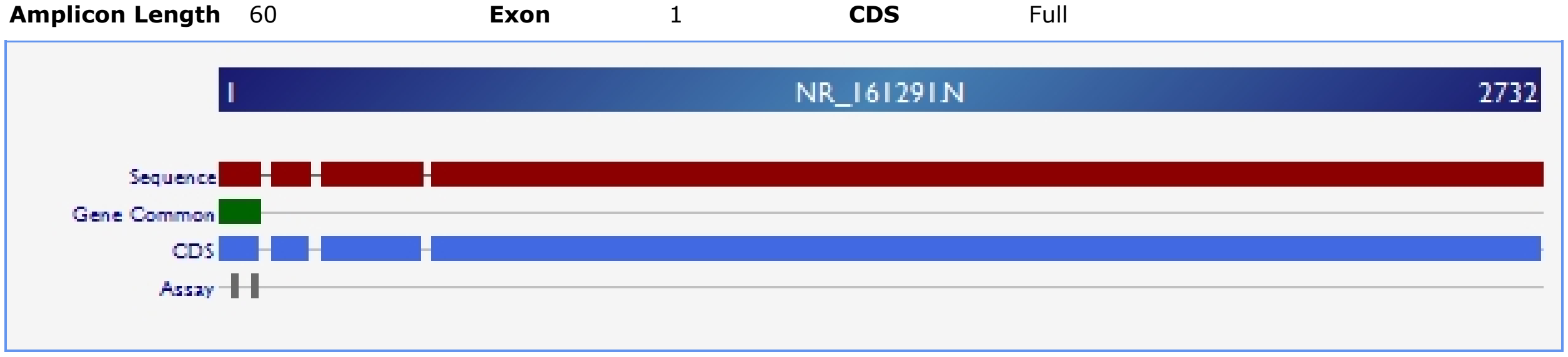

D1

Assay Name DUSP3_17164_i0 **Assay ID** GEA00017164
Gene Symbol DUSP3 **Entrez Gene ID** 1845 **Organism** Homo sapiens
Official Full Name dual specificity phosphatase 3
Related IDs HGNC:HGNC:3069; Ensembl:ENSG00000108861; MIM:Objectid_id; AllianceGenome:HGNC:3069
Also known as VHR
GO Function MAP kinase phosphatase activity; MAP kinase phosphatase activity; cytoskeletal protein binding; myosin phosphatase activity; phosphatase activity; protein binding; protein kinase binding; protein tyrosine kinase binding; protein tyrosine phosphatase activity; protein tyrosine phosphatase activity; protein tyrosine phosphatase activity; protein tyrosine/serine/threonine phosphatase activity; protein tyrosine/serine/threonine phosphatase activity; protein tyrosine/serine/threonine phosphatase activity; receptor tyrosine kinase binding
Go Process cellular response to epidermal growth factor stimulus; dephosphorylation; negative regulation of ERK1 and ERK2 cascade; negative regulation of ERK1 and ERK2 cascade; negative regulation of JNK cascade; negative regulation of JNK cascade; negative regulation of JNK cascade; negative regulation of MAPK cascade; negative regulation of T cell activation; negative regulation of T cell activation; negative regulation of T cell receptor signaling pathway; negative regulation of T cell receptor signaling pathway; negative regulation of cell migration; negative regulation of chemotaxis; negative regulation of epidermal growth factor receptor signaling pathway; peptidyl-tyrosine dephosphorylation; peptidyl-tyrosine dephosphorylation; peptidyl-tyrosine dephosphorylation involved in inactivation of protein kinase activity; positive regulation of focal adhesion disassembly; positive regulation of mitotic cell cycle; positive regulation of mitotic cell cycle; regulation of focal adhesion assembly
Go Component cytoplasm; cytosol; cytosol; immunological synapse; nucleoplasm; nucleoplasm; nucleus
Gene Isoforms NM_004090.
Blast Isoform Hits NM_004090
Amplicon Length 84 **Exon** 1-2 **CDS** Full

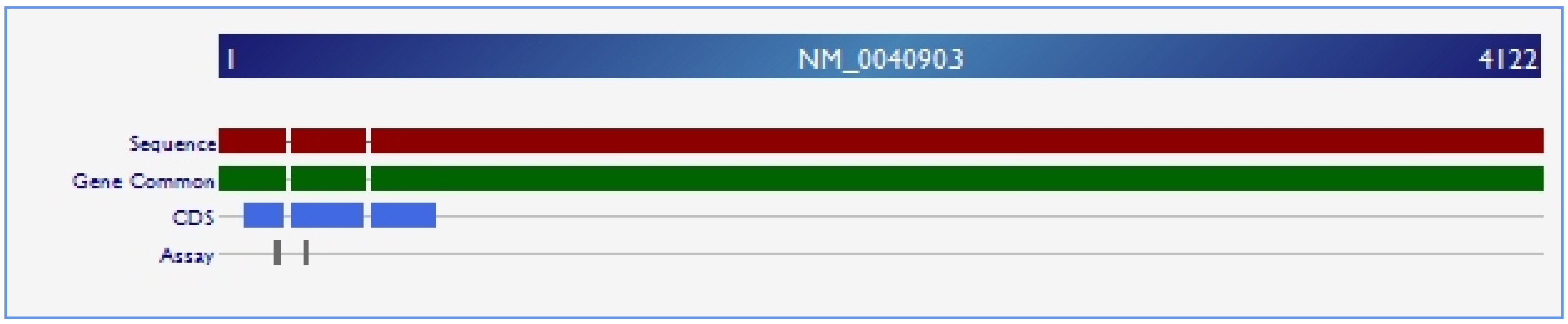

E1

Assay Name FCGR1B_16283_e0 **Assay ID** GEA00016283
Gene Symbol FCGR1B **Entrez Gene ID** 2210 **Organism** Homo sapiens
Official Full Name Fc gamma receptor Ib, pseudogene
Related IDs HGNC:HGNC:3614; Ensembl:ENSG00000291135; MIM:Objectid_id; AllianceGenome:HGNC:3614
Also known as CD34; FCG1; FcRI; CD64b; FCGR1; IGFR1; IGFRB; FCGR1B; FcgammaRIa
GO Function IgG binding; immunoglobulin receptor activity; transmembrane signaling receptor activity
Go Process Fc receptor signaling pathway; adaptive immune response; cell surface receptor signaling pathway; immune response; regulation of immune response
Go Component clathrin-coated endocytic vesicle membrane; early endosome membrane; plasma membrane; plasma membrane
Gene Isoforms NM_001004340.N; NM_001244910.N; NM_001017986.N; NR_045213.N
Blast Isoform Hits NM_001004340; NR_045213; NM_001244910; NM_001017986
Amplicon Length 64 **Exon** 1 **CDS** Partial

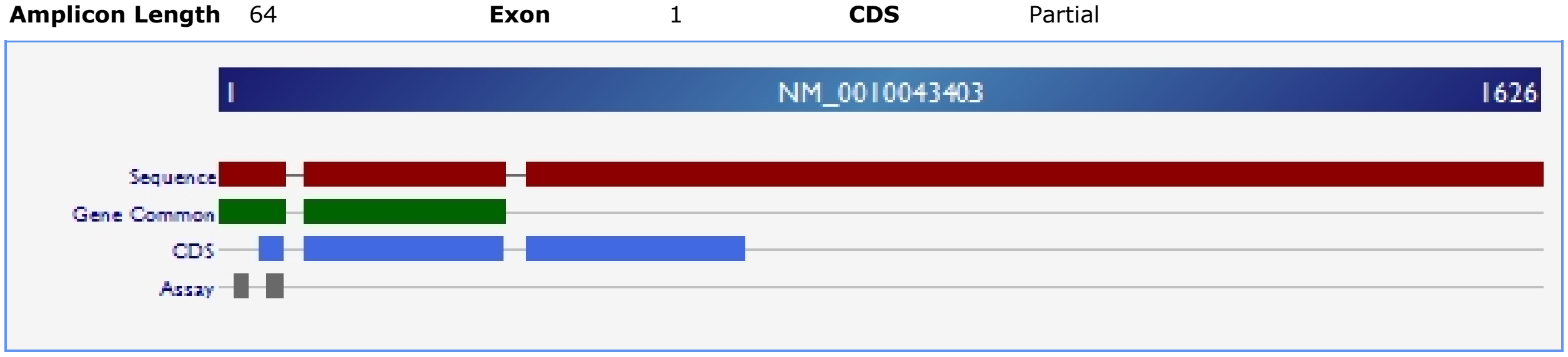

F1

**Assay Name**	FLDM-278021_124252_e0		**Assay ID**	GEP00124252	
**Gene Symbol**	FLDM-278021	**Entrez Gene ID**		**Organism**	Homo sapiens
**Official Full Name**					
**Related IDs**					
**Also known as**					
**GO Function**					
**Go Process**					
**Go Component**					
**Gene Isoforms**					
**Blast Isoform Hits**	FLDM-278021.1				
**Amplicon Length**	76	**Exon**	1	**CDS**	None

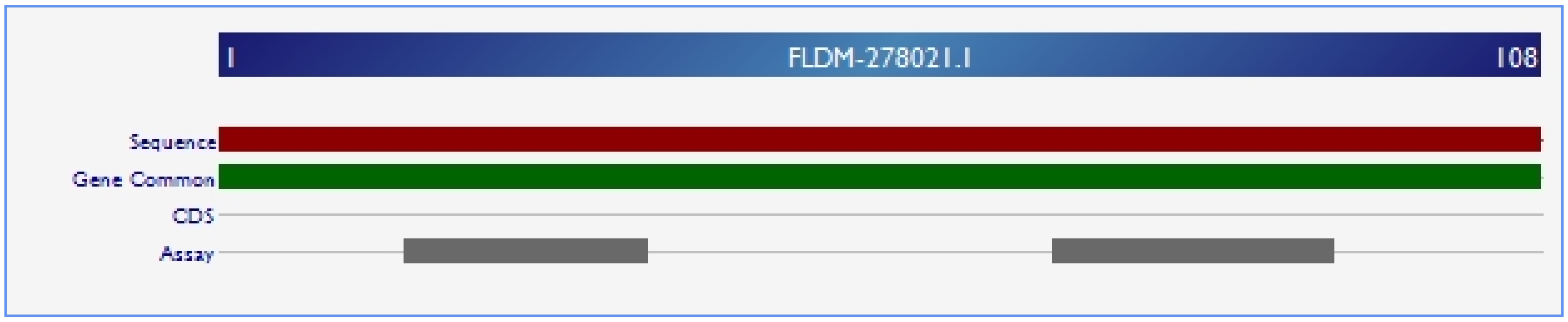

G1

**Assay Name**	FLDM-838169_124255_e0		**Assay ID**	GEP00124255	
**Gene Symbol**	FLDM-838169	**Entrez Gene ID**		**Organism**	Homo sapiens
**Official Full Name**					
**Related IDs**					
**Also known as**					
**GO Function**					
**Go Process**					
**Go Component**					
**Gene Isoforms**					
**Blast Isoform Hits**	FLDM-838169.1				
**Amplicon Length**	99	**Exon**	1	**CDS**	None

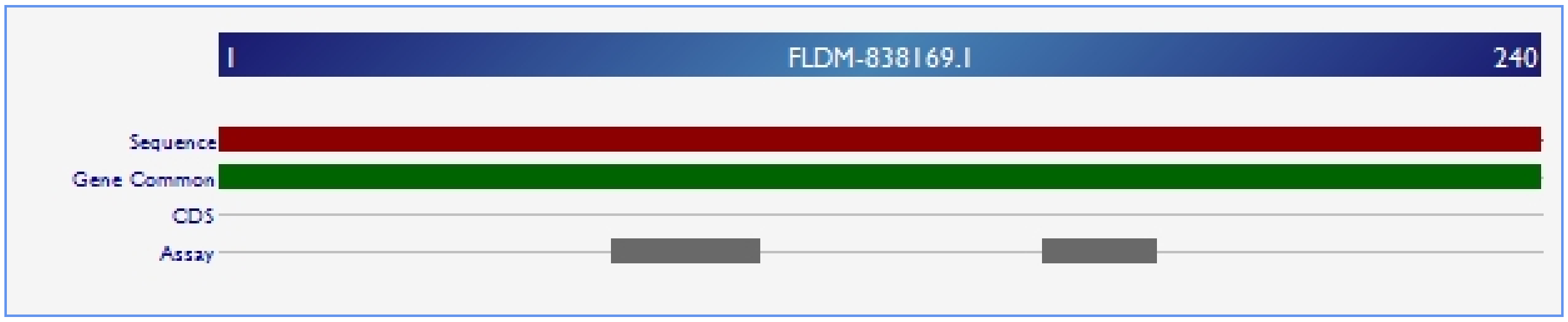

H1

**Assay Name**	FLDM-957085_124253_t-1		**Assay ID**	GEP00124253	
**Gene Symbol**	FLDM-957085	**Entrez Gene ID**		**Organism**	Homo sapiens
**Official Full Name**					
**Related IDs**					
**Also known as**					
**GO Function**					
**Go Process**					
**Go Component**					
**Gene Isoforms**					
**Blast Isoform Hits**	FLDM-957085.1				
**Amplicon Length**	79	**Exon**		**CDS**	None

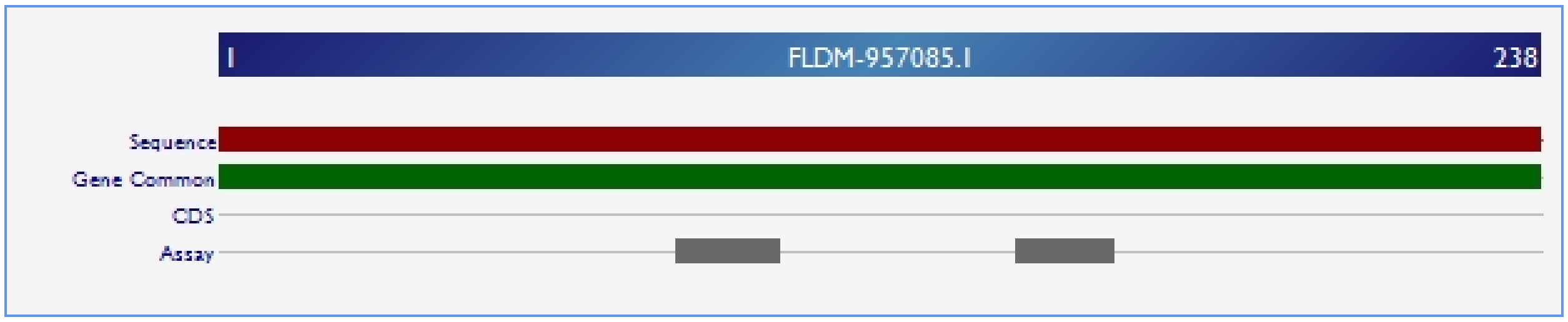

A2

**Assay Name**	FLDM-968540_124250_e0		**Assay ID**	GEP00124250	
**Gene Symbol**	FLDM-968540	**Entrez Gene ID**		**Organism**	Homo sapiens
**Official Full Name**					
**Related IDs**					
**Also known as**					
**GO Function**					
**Go Process**					
**Go Component**					
**Gene Isoforms**					
**Blast Isoform Hits**	FLDM-968540.1				
**Amplicon Length**	77	**Exon**	1	**CDS**	None

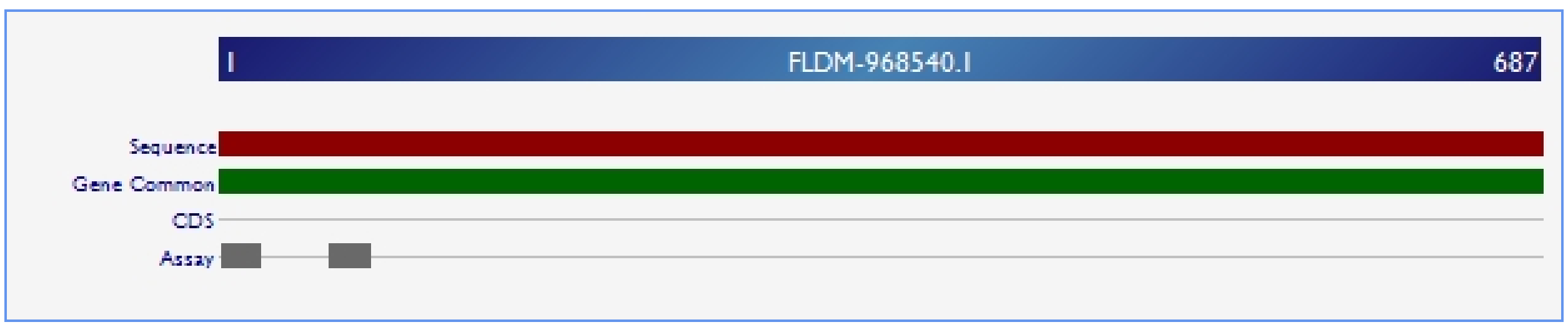

B2

**Assay Name**	FLDM-982892_124249_t-1		**Assay ID**	GEP00124249	
**Gene Symbol**	FLDM-982892	**Entrez Gene ID**		**Organism**	Homo sapiens
**Official Full Name**					
**Related IDs**					
**Also known as**					
**GO Function**					
**Go Process**					
**Go Component**					
**Gene Isoforms**					
**Blast Isoform Hits**	FLDM-982892.1				
**Amplicon Length**	115	**Exon**		**CDS**	None

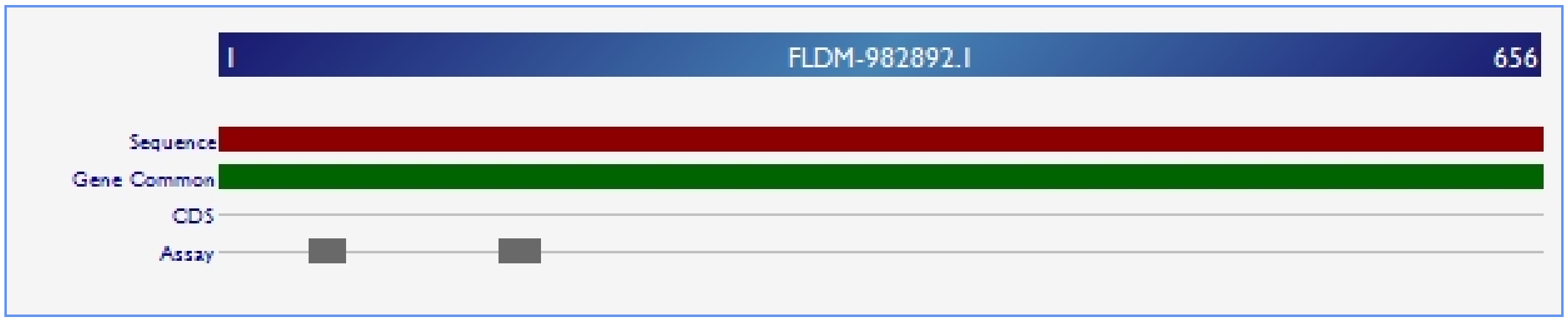

C2

**Assay Name**	GAPDH_93699_i3		**Assay ID**	GEP00093699	
**Gene Symbol**	GAPDH	**Entrez Gene ID**	2597	**Organism**	Homo sapiens
**Official Full Name**	glyceraldehyde-3-phosphate dehydrogenase				
**Related IDs**	HGNC:HGNC:4141; Ensembl:ENSG00000111640; MIM:Objectid_id; AllianceGenome:HGNC:4141				
**Also known as**	G3PD; GAPD; HEL-S-162eP				
**GO Function**	NAD binding; NADP binding; aspartic-type endopeptidase inhibitor activity; disordered domain specific binding; glyceraldehyde-3-phosphate dehydrogenase (NAD+) (phosphorylating) activity; glyceraldehyde-3-phosphate dehydrogenase (NAD+) (phosphorylating) activity; glyceraldehyde-3-phosphate dehydrogenase (NAD+) (phosphorylating) activity; identical protein binding; microtubule binding; peptidyl-cysteine S-nitrosylase activity; protein binding				
**Go Process**	antimicrobial humoral immune response mediated by antimicrobial peptide; cellular response to type II interferon; defense response to fungus; glucose metabolic process; glycolytic process; glycolytic process; killing by host of symbiont cells; killing of cells of another organism; microtubule cytoskeleton organization; negative regulation of endopeptidase activity; negative regulation of translation; negative regulation of translation; neuron apoptotic process; peptidyl-cysteine S-trans-nitrosylation; positive regulation of canonical NF-kappaB signal transduction; positive regulation of cytokine production; positive regulation of type I interferon production; protein stabilization; regulation of macroautophagy				
**Go Component**	GAIT complex; cytoplasm; cytoplasm; cytosol; cytosol; cytosol; cytosol; extracellular exosome; intracellular membrane-bounded organelle; lipid droplet; membrane; microtubule cytoskeleton; nuclear membrane; nucleus; nucleus; nucleus; perinuclear region of cytoplasm; plasma membrane; ribonucleoprotein complex; vesicle				
**Gene Isoforms**	NM_001256799.; NM_001289746.; NM_002046.; NM_001357943.; NR_152150.; NM_001289745.				
**Blast Isoform Hits**	NM_002046; NM_001289746; NM_001256799; NM_001289745; NM_001357943; NR_152150				
**Amplicon Length**	109	**Exon**	4-5	**CDS**	Full

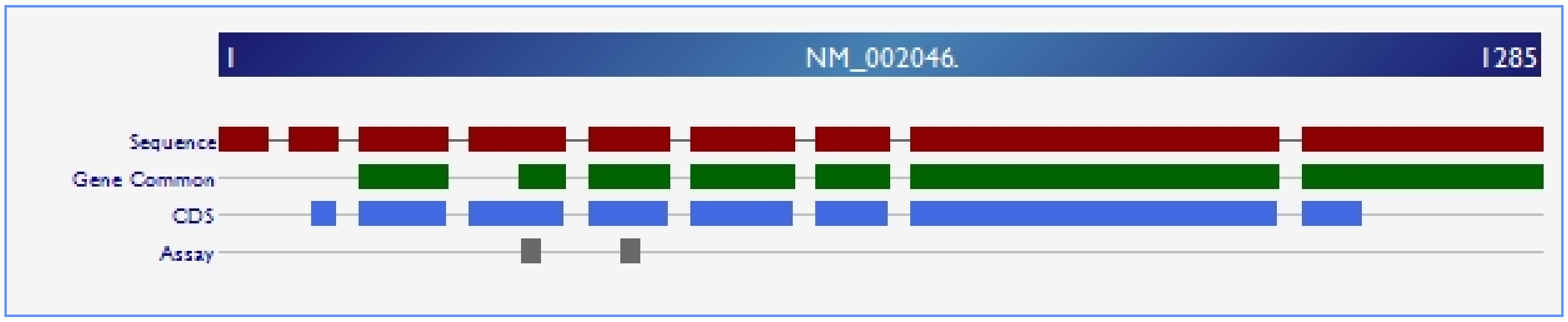

D2

**Assay Name**	GBP2_61748_i9		**Assay ID**	GEP00061748	
**Gene Symbol**	GBP2	**Entrez Gene ID**	2634	**Organism**	Homo sapiens

Official Full Name guanylate binding protein 2

Related IDs HGNC:HGNC:4183; Ensembl:ENSG00000162645; MIM:Objectid_id; AllianceGenome:HGNC:4183

Also known as

GO Function GTP binding; GTPase activity; endopeptidase inhibitor activity; identical protein binding; molecular function inhibitor activity; protein binding; protein homodimerization activity

Go Process activation of innate immune response; cellular response to interleukin-1; cellular response to lipopolysaccharide; cellular response to tumor necrosis factor; cellular response to type II interferon; cellular response to type II interferon; cytolysis in another organism; defense response to Gram-positive bacterium; defense response to bacterium; defense response to protozoan; defense response to virus; immune response; positive regulation of AIM2 inflammasome complex assembly; positive regulation of pyroptosis; protein localization to nucleus

Go Component Golgi apparatus; Golgi apparatus; Golgi membrane; cytoplasm; cytoplasmic vesicle; cytoplasmic vesicle; cytoplasmic vesicle membrane; cytosol; cytosol; nucleoplasm; nucleus; perinuclear region of cytoplasm

Gene Isoforms NM_004120.

Blast Isoform Hits NM_004120

**Amplicon Length**	81	**Exon**	10-11	**CDS**	Full

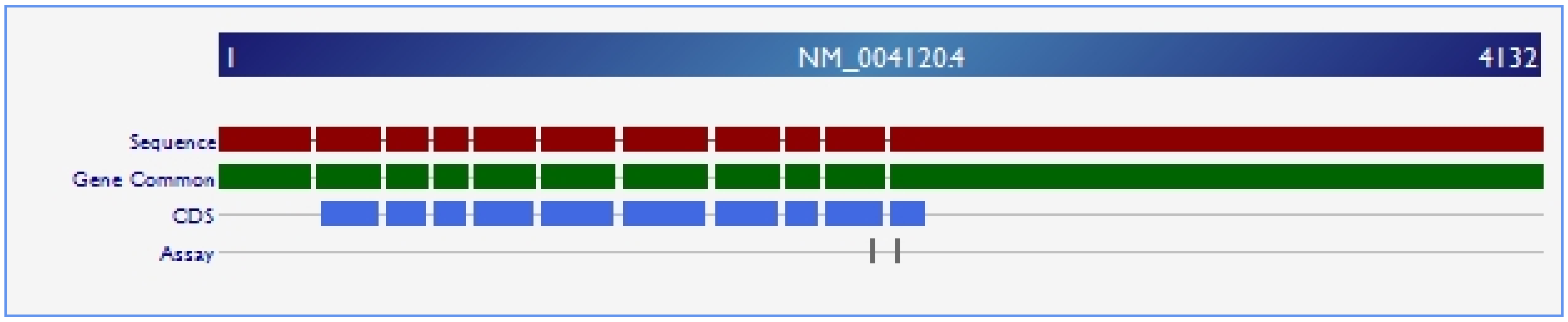

E2

**Assay Name**	GBP5_58588_i2		**Assay ID**	GEP00058588	
**Gene Symbol**	GBP5	**Entrez Gene ID**	115362	**Organism**	Homo sapiens

Official Full Name guanylate binding protein 5

Related IDs HGNC:HGNC:19895; Ensembl:ENSG00000154451; MIM:Objectid_id; AllianceGenome:HGNC:19895

Also known as GBP-5

GO Function GTP binding; GTPase activity; GTPase activity; endopeptidase inhibitor activity; identical protein binding; molecular function inhibitor activity; protein binding; protein homodimerization activity

Go Process activation of innate immune response; cellular response to lipopolysaccharide; cellular response to type II interferon; cellular response to type II interferon; cytolysis in another organism; defense response to bacterium; defense response to virus; inflammatory response; positive regulation of AIM2 inflammasome complex assembly; positive regulation of NLRP3 inflammasome complex assembly; positive regulation of cytokine production involved in inflammatory response; positive regulation of innate immune response; positive regulation of interleukin-1 beta production; positive regulation of interleukin-18 production; positive regulation of pyroptosis; protein homotetramerization; protein localization to Golgi apparatus; protein targeting

Go Component Golgi apparatus; Golgi apparatus; Golgi membrane; cytoplasm; cytoplasmic vesicle; cytoplasmic vesicle membrane; membrane; perinuclear region of cytoplasm; side of membrane; symbiont cell surface

Gene Isoforms NM_001134486.; NM_052942.

Blast Isoform Hits NM_052942; NM_001134486

**Amplicon Length**	75	**Exon**	3-4	**CDS**	Full

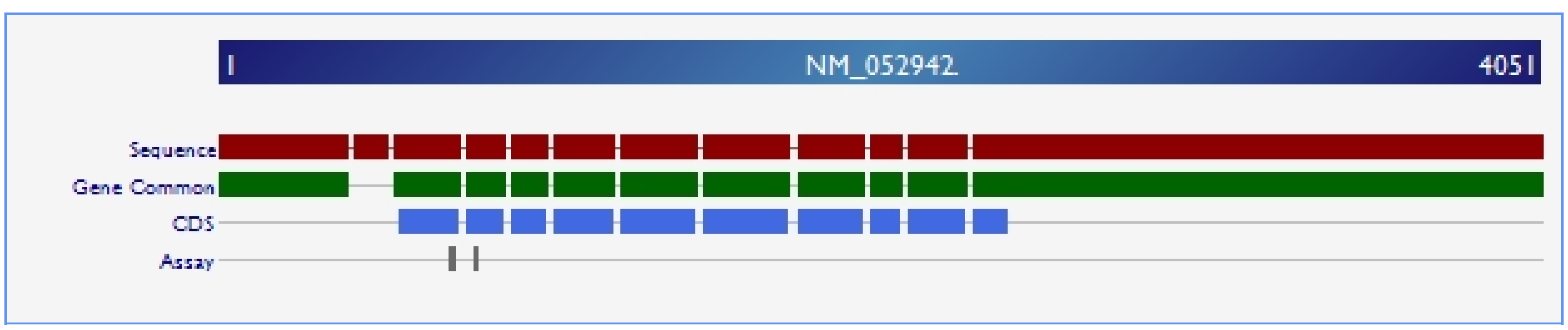

F2

Assay Name HLA-DPB1_58599_i0 **Assay ID** GEP00058599
Gene Symbol HLA-DPB1 **Entrez Gene ID** 3115 **Organism** Homo sapiens
Official Full Name major histocompatibility complex, class II, DP beta 1
Related IDs HGNC:HGNC:4940; Ensembl:ENSG00000223865; MIM:Objectid_id; AllianceGenome:HGNC:4940
Also known as DPB1; HLA-DP; HLA-DPB; HLA-DP1B
GO Function MHC class II protein complex binding; peptide antigen binding; peptide antigen binding; protein binding
Go Process T cell receptor signaling pathway; adaptive immune response; antigen processing and presentation of exogenous peptide antigen via MHC class II; antigen processing and presentation of exogenous peptide antigen via MHC class II; antigen processing and presentation of exogenous peptide antigen via MHC class II; peptide antigen assembly with MHC class II protein complex; positive regulation of T cell activation; positive regulation of T cell activation; positive regulation of T cell proliferation; positive regulation of immune response; positive regulation of type II interferon production
Go Component ER to Golgi transport vesicle membrane; Golgi membrane; MHC class II protein complex; MHC class II protein complex; cell surface; clathrin-coated endocytic vesicle membrane; endocytic vesicle membrane; late endosome membrane; lumenal side of endoplasmic reticulum membrane; lysosomal membrane; lysosomal membrane; membrane; plasma membrane; trans-Golgi network membrane; transport vesicle membrane
Gene Isoforms NM_002121.
Blast Isoform Hits NM_002121.5
Amplicon Length 112 **Exon** 1-2 **CDS** Full

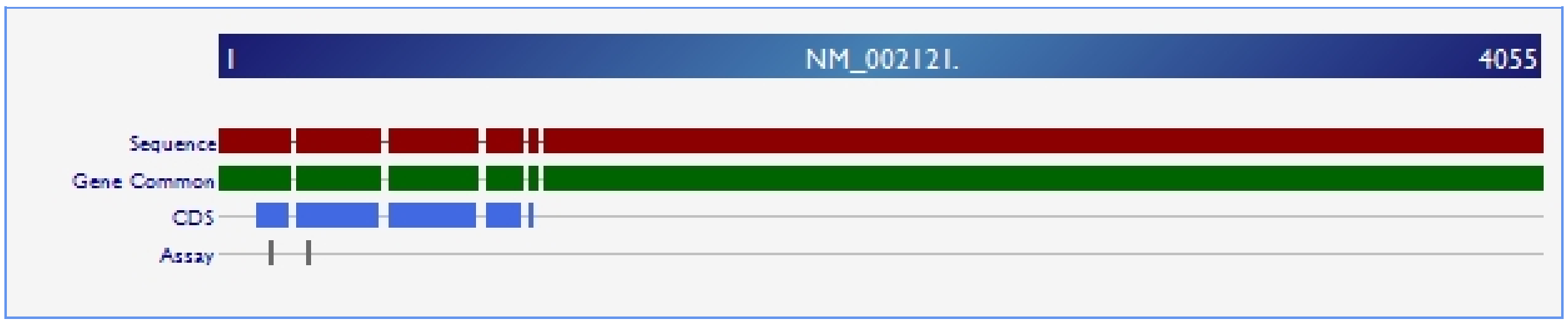

G2

Assay Name KLF2_70436_i1 **Assay ID** GEP00070436
Gene Symbol KLF2 **Entrez Gene ID** 10365 **Organism** Homo sapiens
Official Full Name KLF transcription factor 2
Related IDs HGNC:HGNC:6347; Ensembl:ENSG00000127528; MIM:Objectid_id; AllianceGenome:HGNC:6347
Also known as LKLF
GO Function DNA binding; DNA-binding transcription factor activity; DNA-binding transcription factor activity, RNA polymerase II-specific; DNA-binding transcription factor activity, RNA polymerase II-specific; DNA-binding transcription factor activity, RNA polymerase II-specific; RNA polymerase II cis-regulatory region sequence-specific DNA binding; metal ion binding; protein binding; sequence-specific double-stranded DNA binding
Go Process cell morphogenesis; cellular response to cycloheximide; cellular response to fluid shear stress; cellular response to hydrogen peroxide; cellular response to interleukin-1; cellular response to laminar fluid shear stress; cellular response to peptide; cellular response to tumor necrosis factor; cellular stress response to acid chemical; epigenetic regulation of gene expression; erythrocyte maturation; in utero embryonic development; multicellular organism growth; negative regulation of interleukin-6 production; negative regulation of sprouting angiogenesis; negative regulation of transcription by RNA polymerase II; positive regulation of DNA-templated transcription; positive regulation of nitric oxide biosynthetic process; positive regulation of protein metabolic process; positive regulation of retinoic acid receptor signaling pathway; positive regulation of transcription by RNA polymerase II; positive regulation of transcription by RNA polymerase II; positive regulation of transcription from RNA polymerase II promoter in response to stress; regulation of transcription by RNA polymerase II; type I pneumocyte differentiation; vasodilation
Go Component chromatin; chromatin; nucleus
Gene Isoforms NM_016270.
Blast Isoform Hits NM_016270
Amplicon Length 91 **Exon** 2-3 **CDS** Full

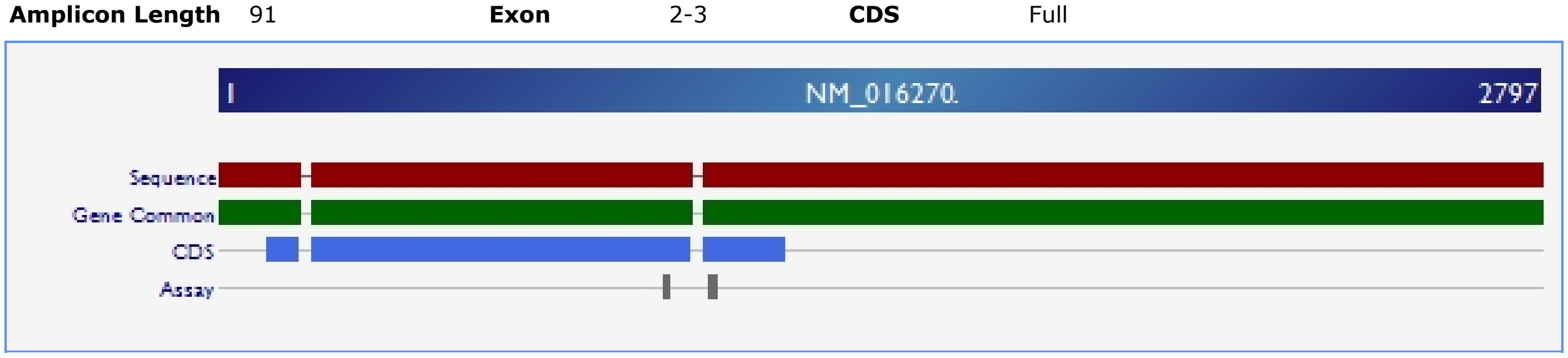

H2

**Assay Name**	METRNL_124238_i2			**Assay ID**	GEP00124238
**Gene Symbol**	METRNL	**Entrez Gene ID**	284207	**Organism**	Homo sapiens

Official Full Name meteorin like, glial cell differentiation regulator

Related IDs HGNC:HGNC:27584; Ensembl:ENSG00000176845; MIM:Objectid_id; AllianceGenome:HGNC:27584

Also known as

GO Function hormone activity; hormone activity; molecular_function

Go Process brown fat cell differentiation; energy homeostasis; energy homeostasis; fat cell differentiation; negative regulation of inflammatory response; positive regulation of brown fat cell differentiation; positive regulation of brown fat cell differentiation; response to cold; response to muscle activity; signal transduction

Go Component extracellular exosome; extracellular space; extracellular space

Gene Isoforms NM_001363853.N; NM_001004431.N

Blast Isoform Hits NM_001004431; NM_001363853

**Amplicon Length**	116	**Exon**	3-4	**CDS**	Full

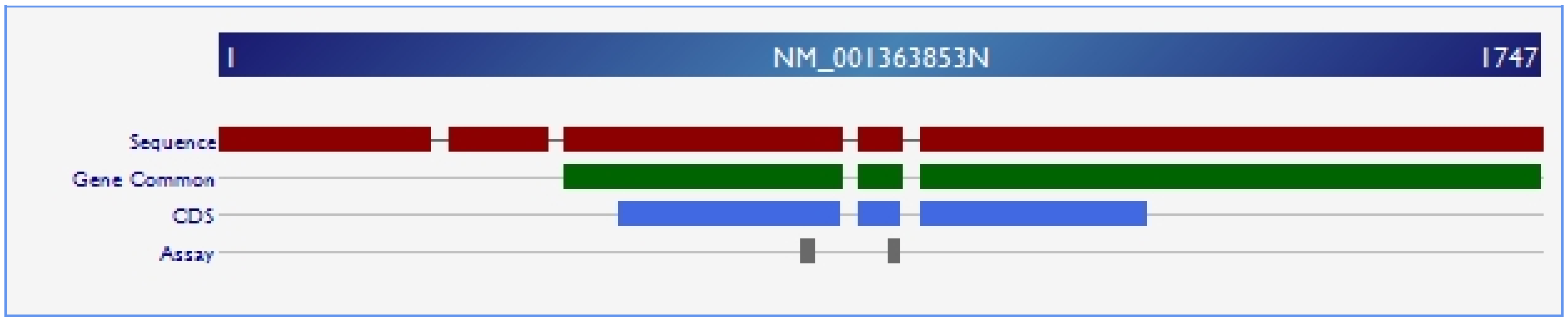

A3

**Assay Name**	RAB11B-AS1_124239_e0			**Assay ID**	GEP00124239
**Gene Symbol**	RAB11B-AS1	**Entrez Gene ID**	100507567	**Organism**	Homo sapiens

Official Full Name RAB11B antisense RNA 1

Related IDs HGNC:HGNC:44178; Ensembl:ENSG00000269386; AllianceGenome:HGNC:44178

Also known as MIR4999HG

GO Function

Go Process

Go Component

Gene Isoforms NR_038237.N

Blast Isoform Hits NR_038237

**Amplicon Length**	88	**Exon**	1	**CDS**	None

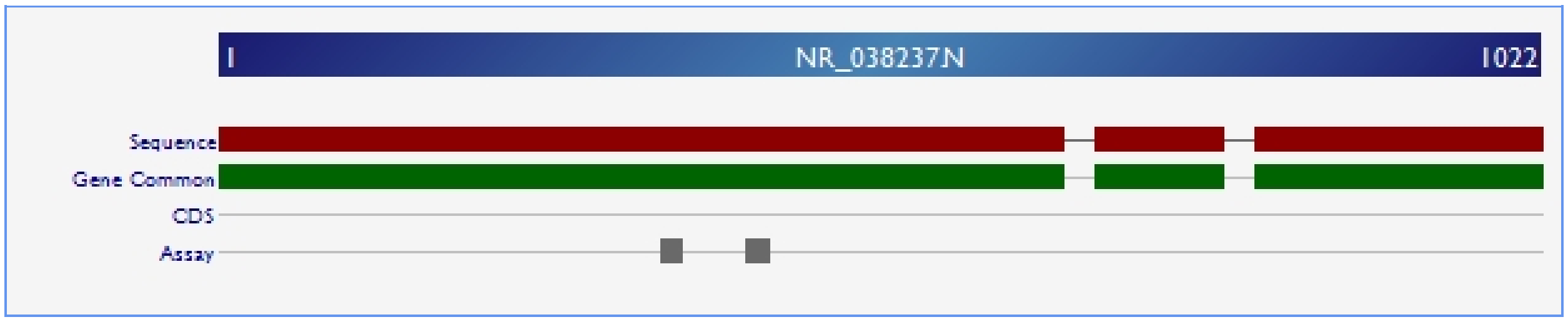

B3

**Assay Name**	SDR39U1_123270_i4		**Assay ID**	GEP00123270	
**Gene Symbol**	SDR39U1	**Entrez Gene ID**	56948	**Organism**	Homo sapiens
**Official Full Name**	short chain dehydrogenase/reductase family 39U member 1				
**Related IDs**	HGNC:HGNC:20275; Ensembl:ENSG00000100445; MIM:Objectid_id; AllianceGenome:HGNC:20275				
**Also known as**	HCDI; C14orf124				
**GO Function**	oxidoreductase activity				
**Go Process**					
**Go Component**	nucleus				
**Gene Isoforms**	NM_001290293.; NM_001308075.; NM_001290292.; NM_020195.				
**Blast Isoform Hits**	NM_001308075; NM_001290292; NM_001290293; NM_020195				
**Amplicon Length**	120	**Exon**	5-6	**CDS**	Full

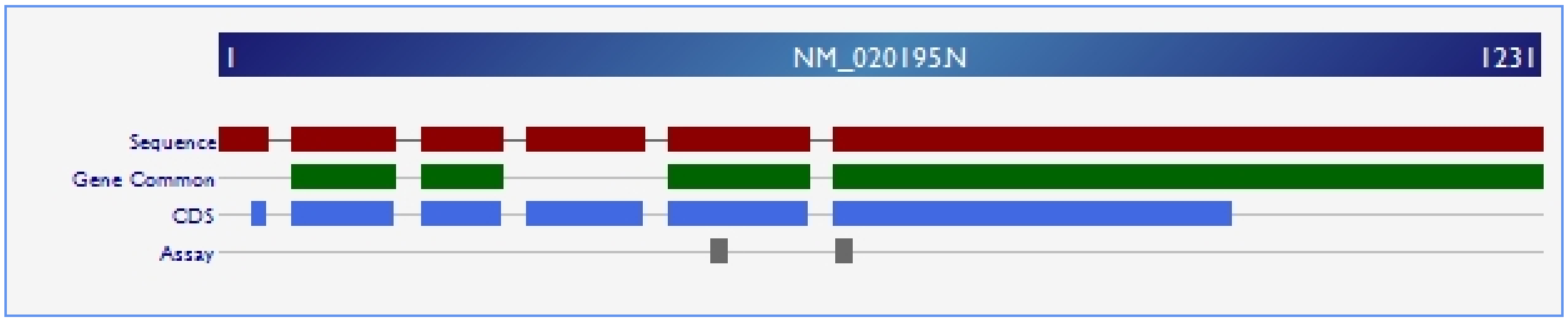

C3

**Assay Name**	SERPING1_55703_i6		**Assay ID**	GEP00055703	
**Gene Symbol**	SERPING1	**Entrez Gene ID**	710	**Organism**	Homo sapiens
**Official Full Name**	serpin family G member 1				
**Related IDs**	HGNC:HGNC:1228; Ensembl:ENSG00000149131; MIM:Objectid_id; AllianceGenome:HGNC:1228				
**Also known as**	C1IN; C1NH; HAE1; HAE2; C1INH				
**GO Function**	protein binding; serine-type endopeptidase inhibitor activity; serine-type endopeptidase inhibitor activity				
**Go Process**	blood circulation; blood coagulation; complement activation, classical pathway; fibrinolysis; innate immune response; negative regulation of complement activation, lectin pathway; negative regulation of endopeptidase activity				
**Go Component**	blood microparticle; collagen-containing extracellular matrix; endoplasmic reticulum lumen; extracellular exosome; extracellular region; extracellular region; extracellular space; extracellular space; platelet alpha granule lumen				
**Gene Isoforms**	NM_000062.; NM_001032295.				
**Blast Isoform Hits**	NM_001032295; NM_000062				
**Amplicon Length**	80	**Exon**	7-8	**CDS**	Full

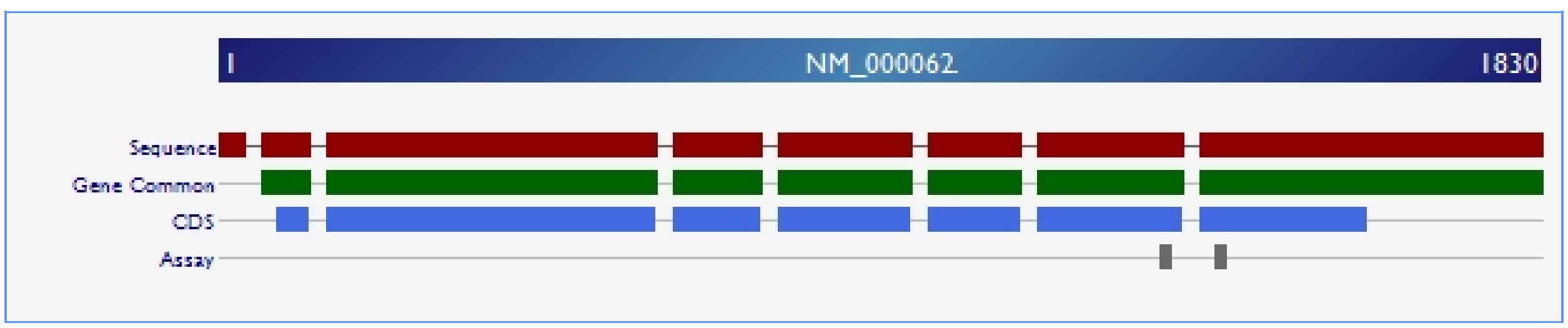

D3

**Assay Name**	TRMT2A_123268_i3		**Assay ID**	GEP00123268	
**Gene Symbol**	TRMT2A	**Entrez Gene ID**	27037	**Organism**	Homo sapiens

Official Full Name tRNA methyltransferase 2 homolog A
Related IDs HGNC:HGNC:24974; Ensembl:ENSG00000099899; MIM:Objectid_id; AllianceGenome:HGNC:24974
Also known as HTF9C
GO Function C-methyltransferase activity; RNA binding; S-adenosylmethionine-dependent tRNA (m5U54) methyltransferase activity; protein binding
Go Process mRNA processing; tRNA methylation
Go Component cytosol
Gene Isoforms NM_182984.; NM_001331039.; NM_022727.; NM_001257994.
Blast Isoform Hits NM_022727; NM_182984; NM_001257994; NM_001331039
Amplicon Length 81 **Exon** 4-5 **CDS** Full

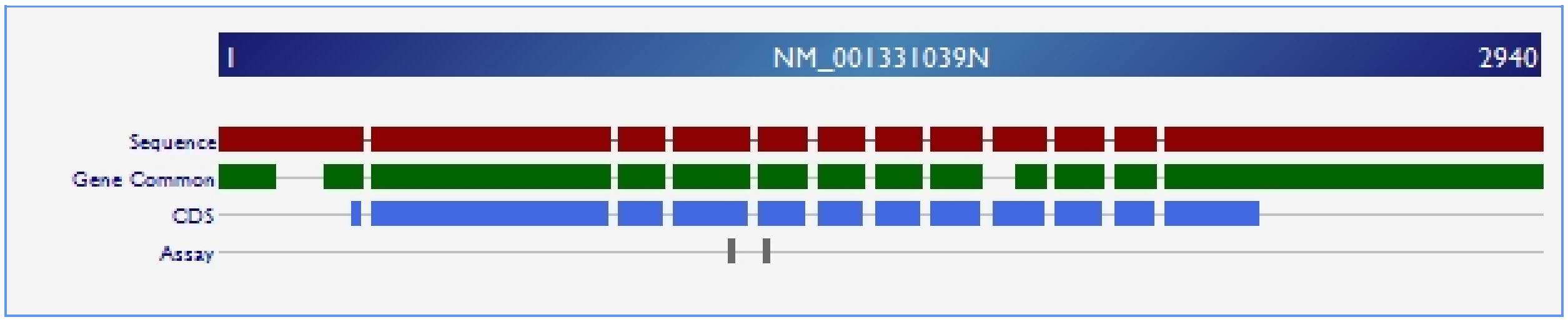

E3

**Assay Name**	TUBGCP6_124243_i3		**Assay ID**	GEP00124243	
**Gene Symbol**	TUBGCP6	**Entrez Gene ID**	85378	**Organism**	Homo sapiens

Official Full Name tubulin gamma complex component 6
Related IDs HGNC:HGNC:18127; Ensembl:ENSG00000128159; MIM:Objectid_id; AllianceGenome:HGNC:18127
Also known as GCP6; GCP-6; MCCRP; MCCRP1; MCPHCR
GO Function gamma-tubulin binding; microtubule binding; microtubule minus-end binding
Go Process cytoplasmic microtubule organization; meiotic cell cycle; microtubule nucleation; microtubule nucleation; mitotic cell cycle; spindle assembly
Go Component centrosome; centrosome; cytosol; equatorial microtubule organizing center; gamma-tubulin complex; gamma-tubulin ring complex; membrane; microtubule; spindle pole
Gene Isoforms NM_020461.
Blast Isoform Hits NM_020461
Amplicon Length 77 **Exon** 4-5 **CDS** Full

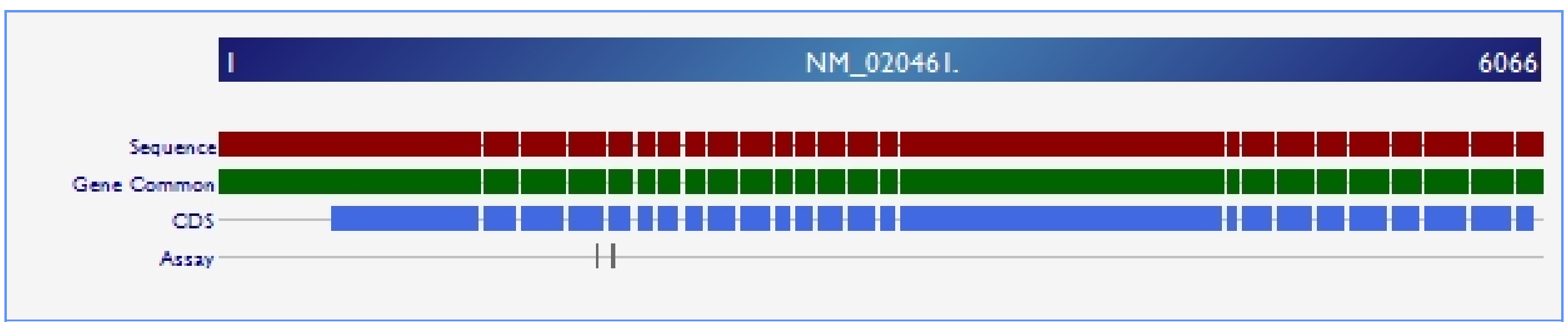

F3

**Assay Name**	UACA_124240_i3		**Assay ID**	GEP00124240	
**Gene Symbol**	UACA	**Entrez Gene ID**	55075	**Organism**	Homo sapiens

Official Full Name uveal autoantigen with coiled-coil domains and ankyrin repeats

Related IDs HGNC:HGNC:15947; Ensembl:ENSG00000137831; MIM:Objectid_id; AllianceGenome:HGNC:15947

Also known as NUCLING

GO Function actin binding; molecular_function; protein binding

Go Process biological_process; negative regulation of non-canonical NF-kappaB signal transduction; positive regulation of cysteine-type endopeptidase activity involved in apoptotic process

Go Component cytoskeleton; cytosol; cytosol; extracellular exosome; extracellular region; nucleoplasm; nucleus

Gene Isoforms NM_001008224.N; NM_018003.N

Blast Isoform Hits NM_018003; NM_001008224

Amplicon Length 87 **Exon** 4-5 **CDS** Full

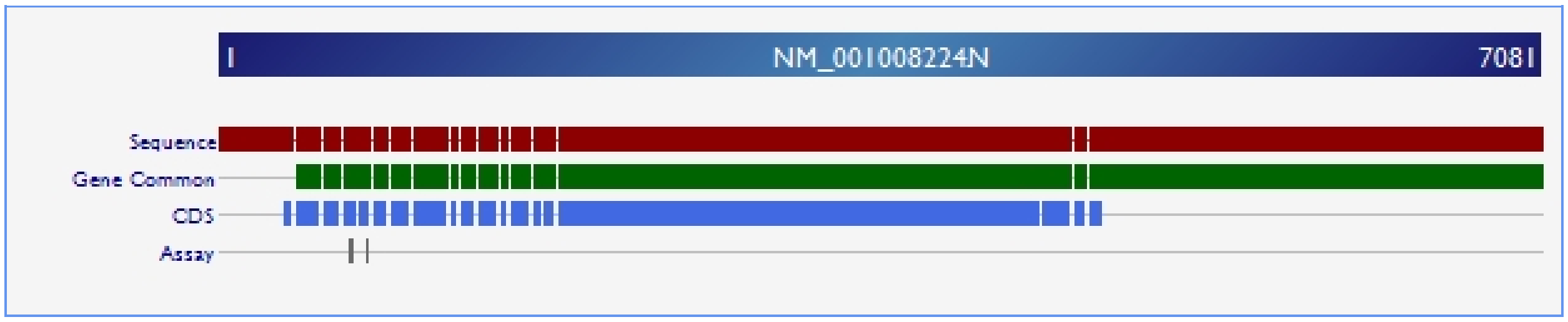

Delta Gene™

Assay Performance Results

JOB ID:	22433_FDGP_23.D1.1	TARGET SPECIES:	Homo sapiens

Results Explanation:
I) Assay performance was evaluated via serial dilution of a commercial reference sample.
II) The ΔCT (or Cq) was calculated for all pairwise combinations of assays in this request and the slope of ΔCT versus log2 template dilution calculated and averaged.
III) The mean slope of ΔCT versus template concentration for cDNA templates indicates relative error in measuring 2-fold changes for the given assay compared to the other assays in this request. If two assays have equal efficiencies and there is no measurement error, the slope will be zero. Typically, 95% of assays fall in the range -0.1 to 0.1.
IV) NO CALL indicates assays that generated < 3 serial dilution measurements or which typically indicates gene expression too low to be reliably measured in the commercial reference sample.

Reference: Livak KJ, Schmittgen TD. Methods 2001; 25:402-408.

Assay Name (ID)	ΔCT Slope	Assay Name (ID)	ΔCT Slope
GEA00016283	-.135	GEA00017164	.125
GEP00055703	.005	GEP00055772	-.013
GEP00058588	-.013	GEP00058599	-.043
GEP00061748	-.016	GEP00070436	.046
GEP00093699	.080	GEP00123268	.076
GEP00123270	.073	GEP00124238	-.200
GEP00124239	.013	GEP00124240	-.048
GEP00124241	-.020	GEP00124242	.032
GEP00124243	-.027	GEP00124249	No Call
GEP00124250	-.009	GEP00124252	-.019
GEP00124253	-.017	GEP00124255	-.200

© Fluidigm Corporation. All rights reserved. For research use only. www.fluidigm.com

Supplementary material 2 Melting point temperatures were manually inspected and adjusted to exclude unspecific products. The cut-offs were set as follows:

Target name	Assay name	Min	Max
DUSP3	DUSP3_17164_i0	85	87
GBP5	GBP5_58588_i2	80	83
KLF2	KLF2_70436_i1	85	89
FCGR1B	FCGR1B_16283_e0	78	80
GBP2	GBP2_61748_i9	80	83
SDR39U1	SDR39U1_123270_i4	84	88
SERPING1	SERPING1_55703_i6	77	79
TRMT2A	TRMT2A_123268_i3	84	86
TUBGCP6	TUBGCP6_124243_i3	83	85
ABLIM2	ABLIM2_124241_i7	80	83
C20orf197	C20ORF197_124242_e0	82	84
CTC-543D15.3	FLDM-982892_124249_t-1*	60	95
CTD-2503O16.3	FLDM-968540_124250_e0	77	82
HLA-DPB1	HLA-DPB1_58599_i0	84	87
METRNL	METRNL_124238_i2	87	92
RAB11B-AS1	RAB11B-AS1_124239_e0	84	87
RP4-614C10.2	FLDM-957085_124253_t-1	84	87
RNA5SP345	FLDM-278021_124252_e0	79	82
RSU1P1	FLDM-838169_124255_e0	83	86
UACA	UACA_124240_i3	80	82
B2M	B2M_55772_i0	81	85
GAPDH	GAPDH_93699_i3	83	88

*Failed assay.

Supplementary Material 3 Table of clinical characteristics of Secondary TB Cases.

Demography		Mycobacterial exposure (baseline)				Individual risk factors				Symptoms or signs of TB at baseline		TB diagnosis
**Gender**	**Age (years)**	**BCG Scar present**	**Tuberculosis Contact Score**	**QFT**	**TST (mm)**	**HIV test**	**Diabetes**	**Smoking last 6 months**	**Alcohol past 6 months**	**Chest X ray**	**Symptoms**	**Positive Mtb cultures**
Female	5	Yes	13	Positive	12	Negative	No	No	No	Normal	No	6 months
Female	10	Yes	12	Positive	18	not performed	No	No	No	Abnormal, not TB	No	12 months
Female	12	No	8	Positive	7	Negative	No	No	No	Normal	No	6 months
Female	12	Yes	13	Positive	15	not performed	No	No	No	Abnormal, not TB. R/po primary complex	No	12 months
Female	19	No	14	Positive	20	not performed	No	No	No	Normal	No	12 months
Female	37	Yes	13	Indeterminate	10	Negative	No	Yes	No	Normal	No	12 months
Female	40	No	12	Positive	12	not performed	No	Yes	No	Normal	No	12 months
Female	51	Yes	13	Negative	6	not performed	Yes	No	No	Abnormal, not TB	No	12 months
Male	6	Yes	14	Positive	6	Negative	No	No	No	Normal	No	6 months
Male	13	No	12	Positive	16	Negative	No	No	No	Abnormal TB. With mediastinal calcification	Yes, cough >2wks	6 months
Male	42	Yes	7	Positive	8	Negative	No	Yes	Yes	Abnormal TB, with apical infiltrate	No	6 months
Male	55	Yes	7	Positive	8	Negative	Yes	No	No	Normal	No	12 months
